# Defined Microenvironments Trigger In Vitro Gastrulation in Human Pluripotent Stem Cells

**DOI:** 10.1002/advs.202203614

**Published:** 2022-12-15

**Authors:** Pallavi Srivastava, Sara Romanazzo, Chantal Kopecky, Stephanie Nemec, Jake Ireland, Thomas G. Molley, Kang Lin, Pavithra B. Jayathilaka, Elvis Pandzic, Avani Yeola, Vashe Chandrakanthan, John Pimanda, Kristopher Kilian

**Affiliations:** ^1^ School of Chemistry Australian Centre for NanoMedicine University of New South Wales Sydney NSW 2052 Australia; ^2^ School of Biomedical Sciences University of New South Wales Sydney NSW 2052 Australia; ^3^ Adult Cancer Program School of Clinical Medicine, Lowy Cancer Research Centre UNSW Sydney Sydney NSW 2052 Australia; ^4^ School of Materials Science and Engineering University of New South Wales Sydney NSW 2052 Australia; ^5^ Katharina Gaus Light Microscopy Facility Mark Wainwright Analytical Centre University of New South Wales Sydney NSW 2052 Australia; ^6^ Department of Haematology Prince of Wales Hospital Randwick NSW 2031 Australia

**Keywords:** embryogenesis, gastrulation, hydrogel, micropatterning, pluripotent stem cells

## Abstract

Gastrulation is a stage in embryo development where three germ layers arise to dictate the human body plan. In vitro models of gastrulation have been demonstrated by treating pluripotent stem cells with soluble morphogens to trigger differentiation. However, in vivo gastrulation is a multistage process coordinated through feedback between soluble gradients and biophysical forces, with the multipotent epiblast transforming to the primitive streak followed by germ layer segregation. Here, the authors show how constraining pluripotent stem cells to hydrogel islands triggers morphogenesis that mirrors the stages preceding in vivo gastrulation, without the need for exogenous supplements. Within hours of initial seeding, cells display a contractile phenotype at the boundary, which leads to enhanced proliferation, yes‐associated protein (YAP) translocation, epithelial to mesenchymal transition, and emergence of SRY‐box transcription factor 17 (SOX17)^+^ T/BRACHYURY^+^ cells. Molecular profiling and pathway analysis reveals a role for mechanotransduction‐coupled wingless‐type (WNT) signaling in orchestrating differentiation, which bears similarities to processes observed in whole organism models of development. After two days, the colonies form multilayered aggregates, which can be removed for further growth and differentiation. This approach demonstrates how materials alone can initiate gastrulation, thereby providing in vitro models of development and a tool to support organoid bioengineering efforts.

## Graphical abstract

Confining pluripotent stem cells to hydrogel islands initiates epithelial to mesenchymal transition and formation of a primitive streak‐like population, without the addition of soluble triggers. The materials directed morphogenesis is a result of mechanotransduction and wingless type (WNT) signaling, which bears similarities to in vivo models of gastrulation.

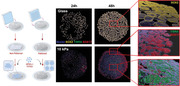

## 1 Introduction

One of the most important events in development is gastrulation, where single layered pluripotent epiblast cells go through a series of carefully regulated cell fate decisions to form the progenitors of the three germ layers.^[^ ^1^ ^]^ Previous studies in mouse have revealed details of the early stages of gastrulation, where cells on the posterior side of the embryo initiate epithelial‐to‐mesenchymal transition (EMT) to form the primitive streak, with subsequent delamination from the epiblast surface after gaining mesenchymal motility.^[^ ^2^ ^]^ The cells in this primitive streak region are positive for the mesodermal marker T/BRACHYURY, and as they ingress inward, they segregate toward endoderm progenitors with endodermal marker SOX17 as the streak extends anteriorly.^[^ ^3^, ^4^ ^]^ These mes‐endodermal cells further proceed to ingress through the primitive streak into the gastrulating embryo.^[^ ^5^ ^]^ The dramatic cellular identity changes in vivo are attributed to an interplay of the transforming growth factor‐beta (TGF*β)*, WNT and fibroblast growth factor (FGF) signaling pathways with their respective antagonists^[^ ^6^ ^]^ and their order of activation and patterns of gene regulation have been well studied.^[^ ^7^ ^]^

Recently, there has been an effort to understand developmental signals in the context of biophysical forces during primitive streak formation; specifically, the local mechanical forces and geometric constraints created by surrounding extra‐embryonic tissues during embryo implantation into the uterine lining.^[^ ^8^ ^]^ Biophysical regulation of the human gastrulation process is difficult to study due to ethical and physiological limitations with handling human embryos during the appearance of the primitive streak, ≈14 days after fertilization.^[^ ^9^ ^]^ Although, current opinion is mixed regarding the importance of the biophysical microenvironment in embryogenesis, it is clear that physical constraints guide cell movements and behavior during streak formation.^[^ ^10^ ^]^ Therefore, in vitro approaches to mimic the embryonic microenvironment, using hydrogels, microcarriers, scaffolds, and other biomaterials, have been employed to study the biophysical basis underlying embryogenesis.^[^ ^11^ ^]^

In vitro models for gastrulation using microengineering have allowed the effect of geometric confinement during tri‐lineage differentiation to be probed.^[^ ^12^, ^13^ ^]^ After bone morphogenic protein‐4 (BMP4) stimulation for 48 h, pluripotent stem cells (PSCs) undergo spatial patterning in response to confinement, which is reminiscent of in vivo processes. Most in vitro studies use rigid glass surfaces (≈3–4 GPa), and exogenous supplements of soluble morphogens to trigger differentiation. In contrast, the signaling gradients in vivo are dynamic, involving local gradients of paracrine and autocrine signals to drive morphogenesis in a confined microenvironment with variable viscoelastic properties.^[^ ^14^ ^]^ Pre‐streak formation events involve actin‐controlled oriented cell movements and deformation on the posterior epiblast, which guide dynamic local gradients of signaling to coordinate primitive streak progression.^[^ ^15^, ^16^ ^]^ Using micropatterning, Gerecht and colleagues demonstrated spatial positioning of T/BRACHYURY^+^ cells using a model of vascular differentiation.^[^ ^17^ ^]^ Similarly, Weaver and colleagues demonstrated multicellular T/BRACHYURY^+^ mesodermal nodes at the colony edges on soft patterned substrates following BMP4 driven differentiation, highlighting the interplay between mechanics and morphogens.^[^ ^18^ ^]^ These in vitro gastruloid mimics show similarities in gene expression and cellular organization with several hallmark features of gastrulation—mes‐endodermal identity (along with comparable downregulation of pluripotent identity), evidence of EMT, and collective cell movement after loss of pluripotency.^[^ ^18^ ^]^

In this article we demonstrate how human pluripotent stem cells spontaneously differentiate into a SOX17^+^ T/BRACHYURY^+^ primitive streak‐like population within 2 days when microconfined on matrix‐conjugated hydrogels. In contrast to previous work where cells required BMP4 stimulation,^[^ ^12^, ^13^ ^]^ here we show how cells microconfined on hydrogels adopt a contractile proliferative phenotype, which leads to epithelial to mesenchymal transition, and spatial differentiation. Gene expression analysis coupled with pathway inhibition studies indicates that mechanotransduction triggers gastrulation on hydrogels through loss of yes‐associated protein ‐ transcriptional enhanced associate domain (YAP‐TEAD) activity and subsequent non‐canonical WNT signaling. Release from confinement and encapsulation in tailored hydrogels results in spatial patterning of differentiation, thereby providing a “materials‐centric” method of forming gastrulation mimics to model embryogenesis.

## 2 Results

### 2.1 Protein‐Conjugated Hydrogels Facilitate Human Induced Pluripotent Stem Cell (iPSCs) Culture

**Figure 1:**
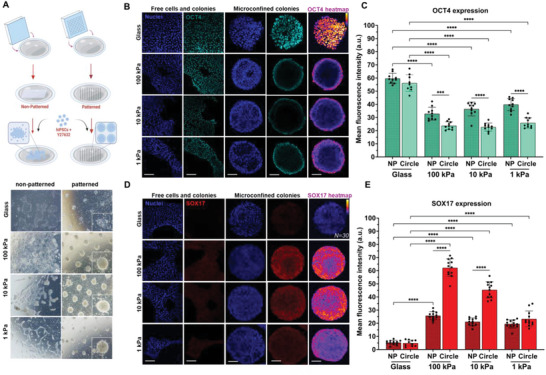
Substrate stiffness directs loss of pluripotency and gain of endodermal identity in microconfined hydrogel culture. A) Schematic of polyacrylamide hydrogel substrate preparation and cell seeding procedure; brightfield images from non‐pattered and patterned (250 µm diameter), glass and hydrogel colonies at 48 h. All images acquired at 4× objective. B) hiPSCs seeded on non‐patterned and patterned glass and hydrogel substrates immunostained for OCT4 at 48 h; right panel—immunofluorescence heatmaps of OCT4 expression. C) Comparison of OCT4 expression intensity across all conditions (*N* = 12). D) hiPSCs seeded on non‐patterned and patterned glass and hydrogel substrates stained for SOX17 at 48 h; right panel—immunofluorescence heatmaps of SOX17 expression and E) Comparison of SOX17 expression across all conditions. One‐way ANOVA, ****p < 0.0001, **p < 0.01, *p < 0.05 (*N* = 12). a.u., arbitrary units. Scale bars: 100 µm.

To assess the behavior of human induced pluripotent stem cells (hiPSCs) on compliant substrates, we chose to work with polyacrylamide (PA) hydrogels with tuneable stiffness that can be further modified using soft lithography to define regions of adhesivity.^[^ ^19^ ^]^ Briefly, the concentration of acrylamide and bis‐acrylamide was varied to create polyacrylamide hydrogel solutions for stiffness of 1, 10, and 100 kPa (tenfold increase spanning physiological tissue^[^ ^20^ ^]^) and then polymerized on chemically modified glass coverslips. The surface of the hydrogel was treated with hydrazine hydrate and then imprinted with an oxidized protein using soft lithography to form the covalent Schiff base using patterned or flat polydimethylsiloxane stamps to mediate cell adhesion on an otherwise inert PA surface. The same stamps were used to assist with physical adsorption of the protein on a clean glass surface to define protein islands. For this study, we had four test groups: protein coated glass, protein patterned glass, protein coated hydrogels and protein patterned hydrogels (hydrogels formulated at 1, 10, and 100 kPa). These groups assessed the response of iPSCs toward confinement alone, stiffness alone, and geometry combined with stiffness, all compared to bare glass controls. While there are differences in the conjugation chemistry between the glass (physisorption) and hydrogel (covalent immobilization), the final density of proteins available for cell adhesion are comparable.^[^ ^19^ ^]^ To screen optimal proteins for iPSC attachment and proliferation, we assayed common matrix proteins involved in in vitro stem cell culture: Engelbreth‐Holm‐Swarm (EHS) Laminin, Laminin‐521, Collagen, Fibronectin, hESC qualified Matrigel, and recombinant human (rh)‐Vitronectin, deposited on substates via soft lithography (Figure S1A, Supporting Information). These trials revealed rh‐Vitronectin to be the most compatible protein which readily facilitated iPSC attachment and proliferation (**Figure** **1**A), as compared to all other tested proteins where high cell death or little to no cell attachment was observed. Since the microcontact patterning process requires oxidation of the printing protein using sodium periodate at room temperature, rh‐Vitronectin, which is generally stable at room temperature proved to be a better alternative to matrigel, which was unstable in supporting patterned iPSC adhesion under these conditions.

Having identified vitronectin as a suitable protein for hiPSC culture, we next asked whether these hydrogel matrices would maintain the pluripotent phenotype. The hiPSCs on glass showed positivity for pluripotency markers (OCT4, SOX2, and NANOG), epithelial marker E‐CADHERIN, and were simultaneously found to be negative for germ layer markers (Figure S1B, Supporting Information). hiPSCs demonstrated healthy attachment on the non‐patterned and patterned hydrogel substrates, with adherent colonies initiating multilayered growth after 48 h with no evidence for spontaneous detachment.

### 2.2 Substrate Properties Alone Guide Human Pluripotent Stem Cell Lineage Specification toward Mes‐Endodermal Identity

With optimized conditions for iPSC culture on hydrogels, we next sought to assess the expression of molecular markers of pluripotency and tri‐lineage differentiation. For geometric confinement, 250 and 500 µm diameter circles were tested. The hiPSCs were dissociated to single cells and seeded at a uniform cell density while being supplemented with Rho‐kinase inhibitor ‐Y‐27632 and allowed to grow for 48 h before fixation (Figure 1A). Optimal cell density was selected based on conditions that foster near confluence on day one.

To evaluate the pluripotency and lineage status of the hiPSCs cultured on these different surfaces, we immunostained the cell populations with the pluripotency marker OCT4 and the endoderm marker SOX17.^[^ ^21^ ^]^ We observed that the cells cultured on glass substrates maintained high levels of uniform OCT4 staining irrespective of pattern (Figure 1B). However, hiPSCs cultured on hydrogels across each stiffness condition demonstrated decreased expression of OCT4 (Figure 1C).

Cells that were microconfined across hydrogels of all three stiffnesses demonstrated ring‐like OCT4 expression at the colony edges, with complete loss of signal in the center (Figure 1B,C). This appearance of an OCT4 annulus in a confined colony was observed previously during cardiac differentiation on patterned glass using CHIR99021,^[^ ^22^ ^]^ which was postulated to be on account of a Wnt signaling–mechanics relationships. Expression of the endoderm marker SOX17 was negligible in colonies on glass, with evidence for modest expression in colonies on non‐patterned hydrogels, indicating a potential role for substrate mechanics in regulating endoderm specification. In contrast, there was a striking upregulation of SOX17 in hydrogel microconfined conditions, with the highest expression observed in cells on 10 and 100 kPa, with decreased expression in cells confined on 1 kPa hydrogels (Figure 1C,D).

**Figure 2:**
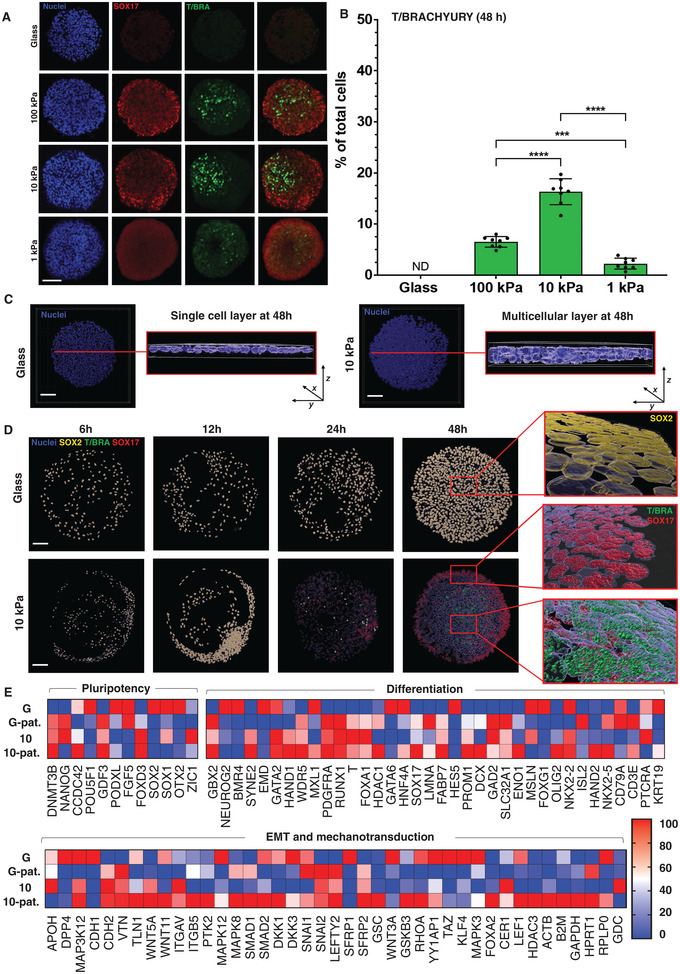
Substrate stiffness directs multilayering and mes‐endodermal population development. A) Immunofluorescence images of colonies on circular patterns on glass and hydrogel substrates, stained for SOX17 and T/BRACHYURY showing mesodermal clustering. B) Percentage of cells expressing T/Brachyury compared to total cells in the colony (*N* = 8). One‐way ANOVA ****p < 0.0001, ***p < 0.001. C) Orthogonal views of surface rendered colonies on glass and 10 kPa hydrogels patterned in 500 µm circles to show monolayered colonies on glass and multilayers on hydrogels. D) Surface rendered images (20×/0.8) of glass and 10 kPa hydrogel patterns at 4 time point; red rectangles depict representative high‐resolution images (63×/1.4), stained with SOX2, SOX17 and T/BRACHYURY. E) Quantitative PCR heatmap for all 4 samples G—glass, G‐pat—glass patterned with 500 µm circles, 10—10 kPa non‐patterned, and 10‐pat—10 kPa patterned with 500 µm circles (smallest and highest value in each data set normalized to 0 and 100, respectively). Scale bars = 100 µm.

Matrix softness has been shown to favor endodermal differentiation.^[^ ^23^, ^24^ ^]^ The SOX17 expression in colonies confined to micropatterned hydrogels was observed as early as 12 h after seeding (Figure S2, Supporting Information), on both 250 and 500 µm diameter micropatterned hydrogels, with the majority of the cells co‐expressing the endodermal marker FOXA2 (Figure S3, Supporting Information). Since we observed loss of pluripotency with gain of endodermal cells on account of hydrogel properties alone, we sought to evaluate the potential for other differentiation outcomes. Co‐staining for T/BRACHYURY demonstrates a multilayered SOX17^+^ structure with a small T/BRACHYURY^+^ mesodermal cell cluster in the centers of both 250 µm colonies (**Figure** **2**A) and 500 µm colonies (Figure S4A, Supporting Information), in more than 50% of the replicates (Figure 2B). All patterned hydrogel samples displayed distinct puncta for T/BRACHYURY within cell nuclei (Figure 2D and Figure S4B, Supporting Information). Expression of T/BRACHYURY^+^ was only evident within cells microconfined on hydrogels, with the highest frequency of distinct central T/BRACHYURY^+^ populations occurring in the 500 µm 10 kPa condition (Figure S4A, Supporting Information). We therefore selected the conditions that showed the highest fraction of cells expressing both SOX17 and T/BRACHYURY, which were the 10 kPa hydrogels patterned with 500 µm circles, for subsequent experiments alongside uniform and patterned 500 µm circles on glass. In addition to the loss of SOX2, a marker for pluripotency and ectoderm, we also stained for the ectodermal marker SOX1 and observed negligible staining (Figure S5, Supporting Information). This indicates that confinement on hydrogels is predominantly initiating mes‐endodermal differentiation, the first stage of gastrulation where the primitive streak is formed. To rule out artifacts associated with reprogrammed cells, we cultured H9 human embryonic stem cells (hESC) under the same conditions and observed a similar trend in expression of lineage markers on 10 kPa hydrogels (Figure S6A–C, Supporting Information).

To better understand how microconfinement on hydrogels is initiating gastrulation, we used high resolution imaging of molecular markers at 6, 12, 24, and 48 h. Three‐dimensional rendered images reveal the cells on glass patterns to be monolayered throughout the experiment, while the cells cultured on hydrogel substrates show progressive multilayering over the course of 48 h (Figure 2C). The initially pluripotent populations show nearly complete loss of SOX2 by 24 h, with uniform SOX17 staining at 24 h and appearance of the punctate T/BRACHYURY^+^ staining occurring between 24 and 48 h (Figure 2D). High resolution imaging demonstrates single and double positive SOX17 and T/BRACHYURY stains, with some indication of 3D structure where the majority of the SOX17+ cells reside at the basal layer while the T/BRACHYURY+ cells reside in the center and top (Figure S7, Supporting Information). To support our immunofluorescence result, we selected our optimal hydrogel condition (10 kPa) compared to glass for quantitative PCR to assess differences in transcript expression on account of substrate stiffness, geometric confinement, and both (Figure 2E; Tables S3–S7). A decrease in the expression level of pluripotency gene OCT4 (POU5F1) (×1.2) and NANOG (×1.5) was observed in the patterned 10 kPa samples compared to glass controls, aligning with the immunofluorescence results. Mes‐endodermal markers SOX17 (×248) and T/BRACHYURY (×32) were considerably higher in the patterned 10 kPa samples, whereas other meso‐ and endodermal markers like PDGFRA (×2), RUNX1 (×2) and FOXA2 (×8) showed a modest increase. Moreover, the primitive streak organizer‐specific transcription factor GOOSECOID (GSC) (×5) was elevated in cells confined to the 10 kPa hydrogels. GSC has been shown to be expressed in the human organizer region, that actively regulates EMT and formation of mes‐endodermal streak.^[^ ^25^ ^]^ A 15× decrease in the epithelial marker E‐CADHERIN (CDH1) coinciding with a 2× increase in the mesenchymal gene N‐CADHERIN (CDH2) in the patterned 10 kPa sample is supportive of epithelial to mesenchymal transition (EMT). In addition, a 2× increase in WNT11 suggests a role for planar cell polarity signaling coordinating the observed differentiation. The non‐canonical WNT pathways are involved in orchestrating EMT^[^ ^26^, ^27^ ^]^ with planar cell polarity pathways guiding the mechanical segregation of cells during gastrulation.^[^ ^28^, ^29^ ^]^ To ensure reproducibility, all results include six technical replicates with a minimum of three biological replicates for each experiment.

Previously we demonstrated how changes in perimeter curvature would influence the behavior of microconfined cells, where geometry and stiffness both exerted an influence over cell phenotype in the context of cancer stemness.^[^ ^30^, ^31^ ^]^ To evaluate whether geometry would play a role in the observed differentiation with our microconfined hiPSC colonies, we cultured cells in shapes of the same area approximating a star, flower, square, and capital “I”—where positive and negative curvature and aspect ratio are varied—followed by immunostaining for germ layer markers. After 48 h there were no significant differences between mes‐endodermal marker expression across the shaped colonies, suggesting changes in geometry at the interface does not play a major role in the observed gastrulation‐like morphogenesis (Figure S8, Supporting Information).

### 2.3 Microconfinement Stimulates Cytoskeletal Tension, YAP Translocation, and Epithelial‐to‐Mesenchymal Transition

**Figure 3:**
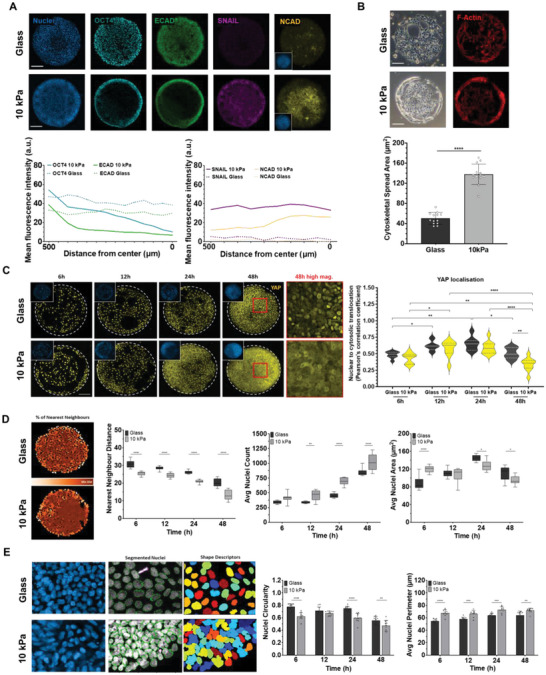
Adhesion stimulates YAP translocation to coordinate epithelial‐to‐mesenchymal transition and differentiation. A) Immunofluorescence images for E‐CAD, N‐CAD, SNAIL, and OCT4 expression across colonies on glass and hydrogel patterns at 48 h. Trace plots quantifying OCT4, E‐CAD, SNAIL, and N‐CAD expression on glass and hydrogels patterns from edge to center. B) Actin spread area comparison at 6 h for glass and hydrogel patterns. C) Time course analysis of adhesion pattern and localization of YAP on glass and hydrogel patterns; YAP and DAPI co‐localization correlation analysis at four time points (Pearson's correlation coefficient) (*N* = 9). D) Representative image depicting the density of nearest neighboring nuclei (darker brown—decreased distance), time course analysis of nearest neighbor distance, average nuclei count and area for glass and hydrogel patterns (*N* = 12). E) Representative DAPI images and nuclei segmentation as primary objects, nuclei shape description on glass and 10 kPa (*N* = 10). Comparisons between two groups was performed using unpaired *t*‐test; comparisons between three or more groups were performed using one or two‐way ANOVA ****p < 0.0001, ***p < 0.001, **p < 0.01, *p < 0.05. a.u., arbitrary units. Scale bars = 100 µm.

Having observed how hydrogel microconfinement alone will trigger mes‐endodermal differentiation, we next sought to investigate how the surface directs this effect. Epiblast cells at the primitive streak region are widely reported to have undergone EMT, to gain their mesenchymal and mes‐endodermal identity before they ingress in the gastrulating embryo.^[^ ^32^ ^]^ Characteristic traits associated with this transformation include increased proliferation, cytoskeletal tension, and changes in cell and nuclear morphology.^[^ ^33^ ^]^ We immunostained microconfined colonies on 10 kPa hydrogel and glass patterns for EMT molecular markers E‐CAD, N‐CAD, and SNAIL, along with OCT4 to gauge pluripotency. As before, we observed OCT4 expression being restricted toward the colony edges with decreased expression toward the center (**Figure** **3**A and Figure S9, Supporting Information). At the same time, there is a loss of E‐CAD in the colony centers, suggesting an EMT prone region starting inward from the periphery. In conjunction with loss of E‐CAD, the population confined on hydrogels expresses uniform SNAIL and N‐CAD expression across the colony. This E‐CAD to N‐CAD switch is considered a prime indicator of cells undergoing EMT and is considered crucial for specification of the primitive streak and other embryogenesis events.^[^ ^34^ ^]^ In comparison, E‐CAD and OCT4 show uniform expression throughout the colonies on glass patterns, with no expression of SNAIL (Figure 3A). Moreover, E‐CAD expression was maintained in colonies across non‐patterned surfaces, with only a few regions showing discontinuous expression (Figure S7B, Supporting Information). This means that only the micropatterned hydrogels are leading to EMT and mes‐endoderm differentiation. We observed a region at the colony edge with a SNAIL^+^ OCT4^+^ population, which is also the region with highest SOX17 expression. We propose that this overlap mimics an early stage in embryo development where SNAIL is reported to control EMT at the epiblast via downregulation of E‐CAD.^[^ ^35^ ^]^ Similar results of discontinuous E‐CAD expression were found using the smaller 250 µm patterns (Figure S10A, Supporting Information). The E‐CAD to N‐CAD switch and concurrent SNAIL expression indicates EMT as a morphogenetic process that correlates with endodermal/mesodermal identity in the microconfined colonies on hydrogels.

Next, we analyzed differences in cell adhesion and proliferation over time in our microconfined cultures with the same initial seeding density. After initial seeding, cells encircle the border and adopt an elongated contractile morphology (Figure S2, Supporting Information) with elevated F‐actin and higher spread area as early as 6 h after seeding, compared to cells in the interior of the pattern or those constrained in glass patterns (Figure 3B). Over time, these contractile cells proliferate to fill the pattern which coincides with lessening of the observed cytoskeletal tension. Confined cells show significantly higher proliferation on the hydrogel patterns compared to glass, which at first glance is counterintuitive since soft matrices have been shown to limit cell proliferation. However, ROCK inhibition with Y27632 has been reported to promote cell proliferation on soft matrices and suppress proliferation on glass through modulating actomyosin contractility.^[^ ^36^ ^]^ This is also apparent through a decreasing distance between nearest neighbors’ analysis (Figure 3D). A core protein in sensing matrix stiffness, and a driver of pluripotency maintenance, is the yes‐associated protein (YAP).^[^ ^37^ ^]^ YAP activity is involved in controlling the expression of genes associated with the anterior primitive streak^[^ ^38^ ^]^ and its deactivation has been indicated in coordinating mes‐endodermal germ layer patterning, while suppressing ectodermal differentiation, during in vitro BMP4 driven differentiation.^[^ ^39^ ^]^ To evaluate YAP localization, we used a custom MATLAB script to evaluate nuclear to cytosolic translocation with a Pearson's correlation coefficient, wherein the co‐localization of a nuclear stain (DAPI) and YAP signal would shift toward +1 if co‐localized, and dip toward −1 if not. YAP expression is predominantly nuclear within the first 12 h, which gradually translocates to the cytoplasm as the cell density increases in the colony (Figure 3C). During this time course we also measured changes in nuclear geometry, where variations in morphology has been demonstrated during EMT, differentiation and de‐differentiation.^[^ ^40^, ^41^ ^]^ Cells confined on 10 kPa hydrogels produce more elongated nuclei with increased nuclear perimeter and decreased circularity over time compared to cells confined on glass (Figure 3D,E). We also performed a zonal analysis to evaluate nuclear changes between the border and interior of the patterns. We see an increase in nuclear area for cells in the center of the hydrogel patterns as early as 6 h, with no significant regional change in nuclear morphometrics for cells adherent on glass islands (Figure S11, Supporting Information). There was no discernible change in cytoplasmic volume across conditions. There are several in vivo studies which demonstrate that cells on the posterior side of the developing embryo undergo a region of dramatic cell movements and intercalation resulting in nuclear elongation/extension in the place where the primitive streak later emerges.^[^ ^42^, ^43^ ^]^ Nuclear shape changes have therefore been associated directly with the emergence of the primitive streak. Overall, these results suggest that initial adhesion increases cytoskeletal tension at the boundary and contributes to increased cell proliferation and crowding in the hydrogel colonies. This resulted in altered nuclear morphology, YAP translocation, and EMT. This stepwise morphogenesis is reminiscent of early phases of gastrulation, where the cells of the epiblast become contractile, undergoing EMT during 3D invagination to specify the primitive streak.

### 2.4 Priming Pluripotent Stem Cell Colonies on Hydrogels Augments BMP4 Induced Germ Layer Specification

**Figure 4:**
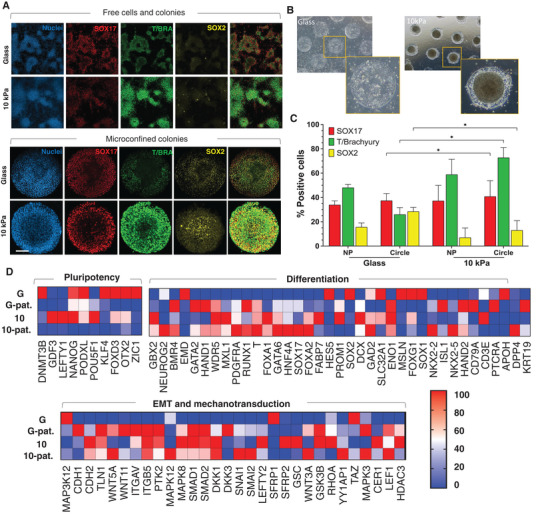
BMP4 treatment augments substrate induced differentiation. A) Colonies on non‐patterned glass versus 10 kPa hydrogels, and on patterned (500 µm) glass versus patterned 10 kPa hydrogels inducted with BMP4 stained for SOX17, T/BRACHYURY and SOX2.  inducted with BMP4 stained for SOX17, T/BRACHYURY, and SOX2. B) Brightfield images of colonies on glass versus hydrogels inducted with BMP4. C) Percentage of positive cells for each lineage compared on glass versus hydrogels, based on the total cells counted in the DAPI image (*N* = 12). arb. = Arbitrary units. ***p < 0.001, **p < 0.01 (Two‐way ANOVA). D) Quantitative PCR heatmap to show gene expression analysis between glass, 500 µm patterned glass, 10 kPa hydrogel and 500 µm 10 kPa hydrogel (smallest and highest value in each data set normalized to 0 and 100, respectively). Scale bars: 100 µm.

Previous work with micropatterned cultures used BMP4 as a soluble morphogen to initiate differentiation.^[^ ^12^, ^13^, ^18^ ^]^ Considering how the micropatterned hydrogel substrate triggers primitive streak‐like morphogenesis, we reasoned that these populations would be more susceptible to differentiation, or bias the population to different outcomes, after induction with BMP4 when compared to the standard condition of culture on glass. To test this, we seeded hiPSCs with BMP4 (50 ng mL^−1^) induction to drive differentiation over 48 h and immunostained for SOX17, T/BRACHYURY, and SOX2. After BMP4 induction, cells cultured on glass show similar spatial partitioning of mes‐endodermal SOX17^+^ T/BRACHYURY^+^ cells with adjacent ectodermal SOX2^+^ (**Figure** **4**) as demonstrated in earlier work.^[^ ^12^ ^]^ The cells seeded on non‐patterned hydrogels showed similar patterns (Figure 4B). Strikingly, the concentric spatial patterning was also observed on micropatterned hydrogels, but with a considerable enhancement leading to a large multilayered cluster in the colony center (Figure 4A,C). Since EMT processes were observed in our cultures without BMP4 (Figure 3), we also immunostained these cultures for E‐CAD which demonstrated a pronounced loss toward the colony edges on glass (Figure S12A, Supporting Information), with SOX17^+^ region at the edges, an enlarged T/BRACHYURY^+^ region that extended toward the center of the colony, and a diminished SOX2^+^ population in the center observed for colonies cultured in confinement on hydrogels compared to glass patterns (Figure 4C). These trends in E‐CAD expression correspond directly with the expression of markers associated with differentiation. The area and perimeter of the differentiated cell nuclei was considerably lower in clustered (differentiated) cells compared to spread cells (undifferentiated) on BMP4 treated cells on glass (Figure S12B, Supporting Information), serving as further evidence for differentiation.

We also performed quantitative PCR for the BMP4 treated cells (Figure 4D). Consistent with the immunofluorescence results for microconfined colonies on hydrogels, we observed decreased expression of pluripotency genes with an increase in differentiation markers toward mes‐endoderm lineages compared to colonies on glass—e.g., endoderm: SOX17 (×340), FOXA2 (×247), and GATA6 (×592), and mesoderm: T/BRACHYURY (×43), RUNX1 (×33), and MIXL1 (×50). We also observed increased expression of EMT regulators N‐CAD (CDH2; ×4), SNAIL (SNAI1; ×13); SNAI2 (×163). Microconfinement on hydrogels led to increased expression of vitronectin binding integrin *α*V (×2) and *β*5 (×4) and associated downstream effectors of mechanotransduction, consistent with our observations of cytoskeletal tension and YAP activity guiding morphogenesis. Concurrently, we observe a further increase in non‐canonical WNT5A (×59) and WNT11 (×2) after BMP4 treatment relative to glass, further suggesting a central role for non‐canonical WNT signals, like the planar cell polarity pathway which dictates patterning during embryogenesis in multiple species.^[^ ^10^, ^44^ ^]^ However, further functional studies using specific WNT knockdowns are necessary to verify this mechanism. Together these results suggest that microconfinement on hydrogels enhances EMT which leads to increased differentiation upon treatment with BMP4.

### 2.5 Yes‐Associated Protein (YAP) Localization and Wingless‐Type (WNT) Signaling Contributes to Differentiation in Spatially Confined Microenvironments

Our results suggest that pluripotent stem cells seeded on hydrogel micropatterns initially experience edge stress that increases cytoskeletal tension and proliferation. These proliferating cells undergo EMT as they fill the pattern with concurrent YAP cytoplasmic translocation and differentiation. However, after cells reach confluency and begin to form differentiated multilayers, there remains a OCT4+ population at the interface. This is consistent with a previous report where an OCT4^+^ annulus remained at the high stress colony edges after CHIR mediated WNT activation in PSCs patterned on glass.^[^ ^22^ ^]^ YAP is a transcriptional activator involved in the Hippo pathway, which is a cornerstone of pluripotency maintenance, involving binding interactions with the TEAD transcription factors.^[^ ^45^ ^]^ In the developing embryo, the YAP‐TEAD complex is inactivated when germ layers are specified, leading to cytoplasmic localization. Since we observed YAP translocation to the cytoplasm at the same time as emergence of the SOX17^+^ T/BRACHYURY^+^ population, we asked whether a similar mechanism was occurring in our patterned populations on hydrogels.

**Figure 5:**
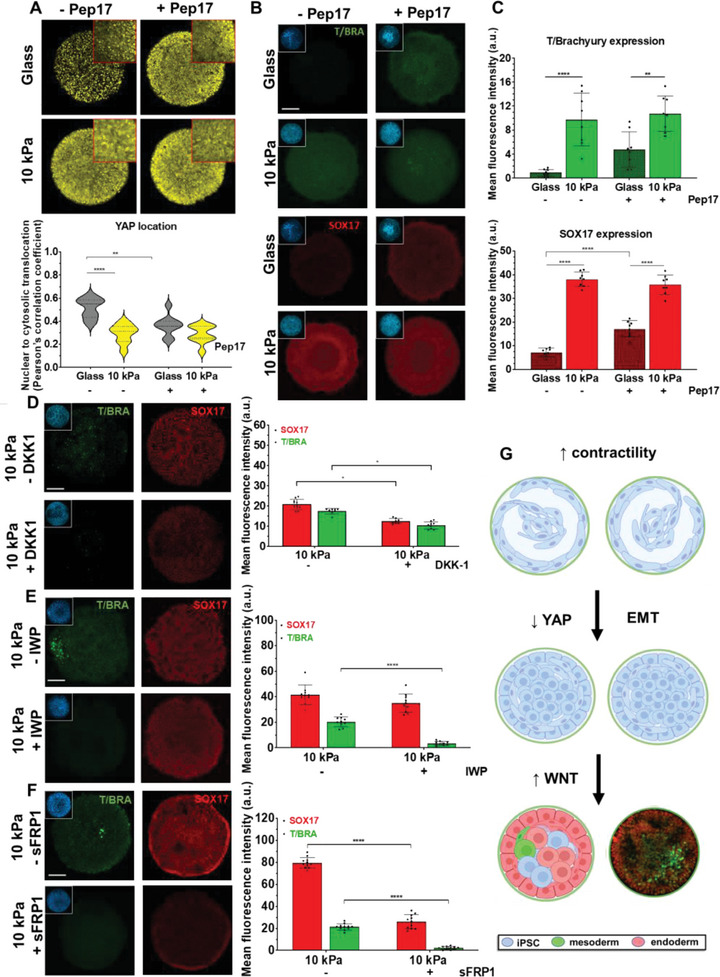
YAP inhibition initiates mes‐endodermal specification and Wnt inhibition disrupts differentiation. A) Immunofluorescence images of glass and hydrogel patterns treated with and without YAP‐TEAD inhibitor peptide17 (±Pep17) stained for YAP; YAP and DAPI co‐localization correlation analysis (Pearson's correlation coefficient) (*N* = 12). B,C) Immunofluorescence images of glass and hydrogel patterns treated with and without Pep17 and stained for SOX17 and T/BRACHYURY with subsequent quantification. D,E,F) Immunofluorescence images of micropatterned iPSCs on hydrogel patterns with and without WNT inhibition (+/‐ DKK1, ±IWP2 and ±sFRP1) and corresponding quantitation. G) Proposed mechanism involving mechanical constraints triggering EMT and mes‐endodermal specification. Comparisons between two groups was performed using unpaired *t*‐test; comparisons between three or more groups were performed using two‐way ANOVA ****p < 0.0001, **p < 0.01, a.u., arbitrary units. Scale bar = 100 µm.

We treated our cultures with a peptide inhibitor that blocks YAP‐TEAD engagement (peptide17).^[^ ^46^ ^]^ Immunostaining our cultures at 24 and 48 h shows that treatment with peptide17 results in more cytoplasmic accumulation of YAP in cells cultured on glass substrates, with no apparent difference in cells cultured on hydrogels (**Figure** **5**A and Figure S13, Supporting Information). Strikingly, the cells patterned on glass treated with peptide begin to express SOX17 and T/BRACHYURY as early as 24 h—with greater than twofold and fivefold increase in both markers at 48 h respectively (Figure 5B)—suggesting that disrupting YAP–TEAD interactions directs mes‐endodermal specification. Treating the cells cultured on patterned hydrogels did not lead to a significant change in mes‐endodermal markers. This is consistent with the hydrogels facilitating YAP cytoplasmic localization and inactivation of YAP‐TEAD signaling.

The YAP inhibition experiments suggest that adhesion to compliant substrates initiates differentiation. However, culture on hydrogels leads to higher expression of mes‐endodermal markers and assembly of multilayered structures, suggesting support from other downstream pathways. The formation of primitive streak in vivo is guided by the activity of TGF*β*/Nodal as well as WNT/*β*‐Catenin pathways.^[^ ^18^, ^47^ ^]^ Since our transcript analysis showed elevated WNT signaling, we supplemented our cultures with the broad spectrum WNT inhibitor IWP2. Treatment with IWP2 leads to complete loss of T/BRACHYURY expression in colonies on the 10 kPa hydrogel. However, the average SOX17 expression remains unchanged compared to vehicle (Figure 5D). This result suggests that mesoderm formation is more sensitive to WNT signaling than endoderm. Our transcript analysis showed increased expression of non‐canonical WNT signals (WNT5A and WNT11), which have previously been shown to coordinate emergence of the primitive streak in model animals.^[^ ^26^, ^29^, ^42^ ^]^ To ascertain a role for canonical and non‐canonical Wnt signaling, we supplemented the culture with soluble Dickkopf‐related protein 1 (DKK1; canonical WNT inhibitor; 100 µg mL^−1^) or secreted frizzled‐related protein 1 (SFRP1; total WNT inhibitor; 5 µg mL^−1^) for 48 h. DKK1 treatment led to partial abrogation of both SOX17 and T/BRACHYURY (Figure 5F), with some expression remaining even at higher doses (Figure S14, Supporting Information). Treatment with SFRP1 which will bind extracellular WNT proteins, thereby impeding both canonical and non‐canonical pathways, led to complete loss of T/BRACHYURY and SOX17 expression (Figure 5E), with maintenance of SOX2 and E‐CAD (Figure S15A,C, Supporting Information). We also probed the effect of other pathway modulators including the GSK*β* antagonist CHIR99021 (CHIR) and ALK4/5/7 inhibitor SB431542 (SB). Treatment with these compounds alone and in combinations led to decreases in the intensity of both markers, albeit to a lesser degree when compared to treatment with IWP2 or SFRP1 (Figure S16, Supporting Information), suggesting a dominant role for WNT signaling in guiding in vitro morphogenesis. Together these results suggest a two‐part mechanism: first, confinement on hydrogels leads to cytoskeletal tension with YAP cytoplasmic translocation; second, subsequent EMT triggers non‐canonical WNT signaling to coordinate differentiation (Figure 5G).

### 2.6 Primed Colonies Can Be Released from the Substrate for Encapsulation

**Figure 6:**
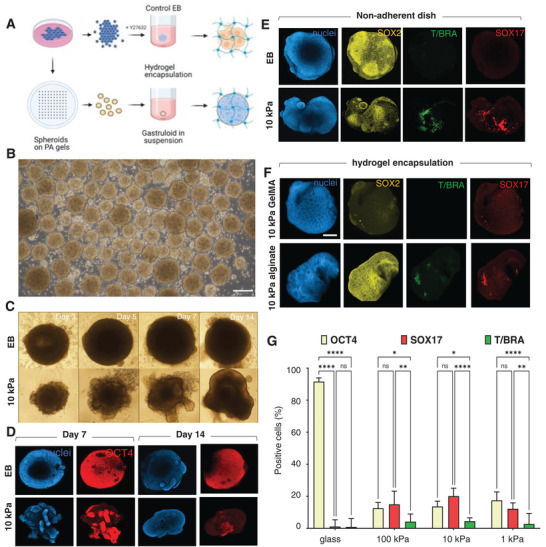
Harvesting differentiating aggregates promotes further growth. A) Schematic for embryoid and gastruloid‐like aggregate formation and encapsulation within hydrogels. B) Brightfield image of pattered aggregates released from the substrate by agitation. C) Brightfield images of embryoid bodies (EB) grown on glass and hydrogel primed gastruloid‐like structures grown in non‐adherent plates. D) Immunofluorescence images of aggregates immunostained for OCT4 after 1 and 2 weeks of culture in non‐adherent plates E) Immunofluorescence images of embryoid bodies and gastruloid‐like structures on non‐adherent plates stained for SOX2, SOX17, and T/BRACHYURY and F) after encapsulation for 14 days in indicated hydrogel biomaterials. G) Percentage of positive area for OCT4, SOX17, and T/BRACHYURY expression in embryoid bodies (Control) and hydrogel cultured gastruloid‐like structures. One‐way ANOVA ****p < 0.0001, *p < 0.05. Scale bar = 100 µm.

Since microconfined hiPSCs on gels promote mes‐endodermal differentiation without exogenous stimulation, we sought to discern subsequent spatial morphology and organization for aggregates harvested from the substrates. After culture for 48 h, primed cultures were lifted off the hydrogels intact and individually cultured for 14 days in non‐adherent conditions. To control for the primed conditions, hiPSCs were seeded in low density non‐adherent plates to form embryoid bodies (**Figure** **6**A. The embryoid bodies show uniform spherical growth while the gastruloid‐like aggregates show an unrestricted structural growth from the periphery over two weeks (Figure 6B,C). At day 7, we observe a distinct loss of OCT4 expression in the growing gastruloid‐like structure compared to embryoid, which is further downregulated by day 14 (Figure 6D. Immunostaining for SOX2 (Ectoderm/Epiblast cells), SOX17 (Endoderm) and T/BRACHYURY (Mesoderm/Primitive Streak) shows loss of OCT4^+^ regions at the interface, with partial SOX2 staining and hotspots of SOX17 and T/BRACHYURY, which is not observed in the embryoid bodies (Figure 6E. The appearance of positional primitive streak‐like populations is reminiscent of early stages of embryonic gastrulation; however, the magnitude and directionality of the outgrowths vary across samples.

The ability to harvest these differentiating colonies through simple agitation provides scope for further processing, including encapsulation in hydrogels. As proof of concept, we encapsulated the gastruloid‐like structures in common biomaterials including gelatin methacryloyl (GelMA), methacrylated hyaluronan (MeHA) and alginate. At day 7, the multi‐lobed structures spread out and became more spherical like the embryoid bodies when confined within photocrosslinked gelatin methacryloyl (GelMa) or methacrylated hyaluronan (MeHa) with gradual loss of pluripotency and primitive streak hotspots (Figure 6F,G. However, encapsulation within non‐covalently stabilized alginate demonstrates elongation of the structure with converging SOX17^+^ and T/BRACHYURY^+^ regions. These results highlight how viscoelastic materials like alginate can accommodate cell reorganization, which may prove useful in future studies of morphogenesis.

To determine if the gastruloid‐like structures would differentiate in vivo and show any germ layer bias, we performed a subcutaneous teratoma assay using immunocompromised SCID mice with embryoids and differentiating aggregates from the 10 kPa hydrogels. There was high variability in growth across all conditions with no discernible trend in size across starting cell types (Figure S17, Supporting Information). To determine if the implanted cells undergo differentiation in vivo, we performed histology scoring to classify structures associated with different germ layers. We observed duct‐like, cartilaginous, bone, loose mesenchyme, smooth muscle, neural rosettes/neuroectoderm, pigmented epithelium, and squamous epithelial‐like structures. While the overall histology scoring indicates no significant difference across the cell types, there were subtle variations in the occurrence of specific structures that may be related to the primed mes‐endodermal population (Figue S17) .

## 3 Discussion

Bioengineered in vitro models have become powerful tools to study relationships between biophysical microenvironments and intracellular signaling pathways that align to specify cell fate in populations of pluripotent stem cells.^[^ ^47^, ^48^ ^]^ In recent years, micropatterned systems have shown how geometric confinement can guide cellular assembly, where triggering differentiation with soluble morphogens has led to in vitro models of embryogenesis,^[^ ^12^, ^13^, ^49^ ^]^ neuroectoderm,^[^ ^50^, ^51^ ^]^ vascular,^[^ ^17^, ^52^ ^]^ and cardiac tissue.^[^ ^22^ ^]^ While these models relied on soluble factors to initiate differentiation, here we demonstrated how pluripotent stem cells micropatterned on deformable substrates initiated in vitro morphogenesis‐like events through interactions with the substrate alone.

After initial attachment to the micropatterned islands on hydrogels, cells adherent to the high stress regions at the periphery developed elongated morphology with high cytoskeletal tension. In contrast to the uniform adhesion and proliferation on glass, cells on the soft hydrogels show enhanced proliferation, loss of nuclear YAP—a characteristic of germ layer specification^[^ ^53^ ^]^—EMT and subsequent differentiation to a mes‐endodermal SOX17^+^ T/BRACHYURY^+^ population. This behavior of contractile cells at the boundary coordinating EMT is reminiscent of behavior in vivo where the pluripotent epiblast adopts a contractile phenotype initiating EMT and formation of the primitive streak.^[^ ^26^, ^42^ ^]^ YAP activity is essential for maintenance of pluripotency in the embryo, and mechanotransduction drives cytoplasmic translocation at the onset of differentiation.^[^ ^39^ ^]^ Moreover, YAP activity is implicated in regulating mes‐endodermal formation at the anterior primitive streak.^[^ ^38^ ^]^ We propose that hydrogel microconfinement provides initial conditions that are analogous to the posterior epiblast, where stress fostered by the pattern boundary promotes contractile cells, proliferation, YAP cytoplasmic translocation, EMT and formation of a spatially confined primitive streak‐like population. Treating micropatterned cultures on glass with an inhibitor of YAP nuclear activity leads to cytoplasmic translocation and initiation of differentiation, thereby supporting YAP localization as a step mediating differentiation.

The cells lining the perimeter of the colonies on hydrogels co‐expressed OCT4, SOX17, E‐CAD and SNAIL, whereas the central part of the colonies showed loss of both OCT4 and E‐CAD but with an increase in expression of SNAIL and mes‐endodermal markers SOX17 and T/BRACHYURY. The co‐expression of OCT4 and SOX17 at the edge suggests partial differentiation consistent with early phases of endoderm specification.^[^ ^54^, ^55^, ^56^ ^]^ Furthermore, the expression of SNAIL suggests the boundary cells retain differentiation ability, where co‐expression of OCT4 and SNAIL has been shown to regulate epiblast exit to facilitate mesoderm differentiation.^[^ ^57^ ^]^ SOX17 is one of the first transcription factors to be expressed in the inner cell mass^[^ ^58^, ^59^ ^]^ and regulates the dynamics of pluripotency and differentiation.^[^ ^60^ ^]^ Moreover, The anterior region of the primitive streak is populated by definitive endodermal population in early and mid‐streak embryonic stages.^[^ ^3^ ^]^ T/BRACHYURY marks the first mesodermal cells that ingress in the primitive streak of a gastrulating embryo.^[^ ^61^, ^62^ ^]^ Confinement of pluripotent stem cells on hydrogels guides expression of SOX17 as early as 12 h, with low‐level transient expression of T/BRACHYURY followed by robust expression in subpopulations after 24 h, leading to a multilayered structure with primitive streak characteristics. This finding conflicts to a degree with the accepted order of differentiation at the onset of gastrulation where T/BRACHYURY^+^ cells precede the SOX17^+^ population. Nevertheless, the appearance and spatiotemporal organization of mixed SOX17^+^ and T/BRACHYURY^+^ cells mimics the emergence of a primitive streak‐like population, with scope for studying early morphogenesis during gastrulation.

There is considerable evidence to support a central role for biophysical cues directing initiation of gastrulation.^[^ ^8^, ^14^ ^]^ Embryonic epiblast is a layer of epithelial cells tightly connected and packed at the interface as the embryo implants itself on the maternal uterine lining.^[^ ^63^ ^]^ At the onset of gastrulation, the cells at the posterior side of the embryo undergo epithelial‐to‐mesenchymal transition (EMT),^[^ ^2^, ^64^ ^]^ a signaling gradient arises between BMP, WNT, and Nodal pathways, and the primitive streak appears at the same EMT‐prone region.^[^ ^1^, ^65^ ^]^ In vivo, the initiation of EMT precedes the appearance of the first multipotent mes‐endodermal progenitors at the primitive streak^[^ ^64^ ^]^ which triggers the epiblast layer toward an intercalating, mesenchymal identity at the onset of gastrulation.^[^ ^42^ ^]^ EMT leads to loss of apical‐basal polarity with concurrent nuclear shape changes that contribute to adhesion patterns and motility during formation of the primitive streak.^[^ ^66^ ^]^ Similarly, we see adoption of a mesenchymal phenotype at the pattern boundary, with distinct changes in cell and nuclear shape as EMT progresses. These microconfined colonies on hydrogels therefore emulate a disk‐like region of the epiblast, where the “junctional‐tension and intercalation” mechanism observed in vivo is replicated by microenvironmental signals from the pattern boundary in vitro, thus catalyzing morphogenesis to a mes‐endodermal population (Figure S18, Supporting Information).

Transcript analysis indicates that non‐canonical WNT signaling, like the planar cell polarity pathway, is considerably upregulated in the hydrogel micropatterned colonies. It is well appreciated that primitive streak cells arise due to endogenously produced WNT ligands.^[^ ^67^, ^68^, ^69^, ^70^ ^]^ WNT signaling controls differentiation during vertebrate development, and the non‐canonical WNT pathways are involved in coordinating EMT^[^ ^26^, ^27^ ^]^ with planar cell polarity guiding the mechanical segregation of cells during gastrulation.^[^ ^28^, ^29^ ^]^ After treating our cultures with small molecule inhibitors of WNT/*β*‐catenin pathways we see some moderate attenuation of mes‐endodermal differentiation. Treatment with the WNT inhibitor IWP2 prevents T/BRACHYURY expression but does not significantly impede SOX17 expression irrespective of concentration. Specific inhibition of Wnt signaling using the soluble protein DKK1, which impedes canonical Wnt binding to frizzled receptors at the membrane, leads to partial attenuation of SOX17 and T/Brachyury expression. However, when using the soluble protein WNT inhibitor SFRP1, which inhibits all WNT signaling in the extracellular space, there is complete abolishment of both markers. This provides direct evidence that non‐canonical WNT signaling is necessary for differentiation to proceed in this system. We propose that the mechanics of the patterned hydrogel signal YAP disengagement and cytoplasmic localization, which initiates EMT processes with endogenous WNT signaling to coordinate the appearance and organization of a mes‐endodermal primitive‐streak like population. However, at this time these relationships are correlative and further work with specific knockdowns are required to isolate the key molecular players responsible for differentiation.

Models of gastrulation have typically used the soluble protein trigger BMP4 to initiate differentiation.^[^ ^12^, ^13^, ^49^ ^]^ In comparison to glass microconfined colonies, treatment of our hydrogel colonies with BMP4 leads to a clear change in the morphology of the aggregates as they differentiate into 3D with distinct clusters of mesodermal cells. Quantification of expression indicates a slight increase in endoderm specification and a large increase in mesoderm, confirming the role of substrate stiffness in enhancing these lineages. Changes at the transcript level are even more striking, where BMP4 treatment leads to 50–100 fold increases in expression of several mes‐endodermal genes and molecular markers of EMT. Together, this suggests that hydrogel culture may prove a method to accentuate morphogenesis on account of exogenous soluble factors, or to eliminate factors during differentiation protocols thereby lowering cost and time and/or improving efficiency.

The ability to foster gastrulation‐like events through the properties of the culture substrate alone opens opportunities to study the relationships between the biophysical microenvironment and early embryogenesis. Colonies that have been primed for two days in microconfinement were released from the substrate and cultured in suspension to see if further patterning would occur in standard conditions for embryoid body growth. Compared to control embryoid bodies which exhibit a uniform morphology with robust staining of pluripotency markers, the primed colonies exhibited clear regions of mes‐endodermal identity which further evolved into multilobed structures. The morphology of these aggregates show similarities to approaches involving small molecule stimulation of suspended embryoid bodies.^[^ ^71^ ^]^ The ease in which the gastruloid‐like structures can be released provides scope for using this technique to prime embryoids for further differentiation, i.e., to support organoid bioengineering efforts, and we demonstrate encapsulation within a panel of hydrogel biomaterials for continued growth. Overall, these finding underscore the importance of materials in nurturing the differentiated phenotype during in vitro differentiation, which draws parallels to the role of the extracellular matrix in orchestrating embryogenesis in vivo.

## 4 Conclusions

Modeling the onset of human gastrulation in vitro through materials properties without exogenous biochemical induction raises numerous opportunities for probing fundamental stages in embryogenesis. The precise control of gastrulation in vivo is afforded by tight coordination of soluble signals with feedback from the biophysical microenvironment. Using microengineered hydrogel substrates, we demonstrate a method where controlling the biophysical microenvironment poises a population of pluripotent stem cells to undergo morphogenesis, with integration of soluble signals to orchestrate gastrulation‐like processes. These findings provide a new avenue for probing the biophysical and biochemical basis of embryogenesis, and a tool to model development for fundamental biology and translational endeavors.

## 5 Experimental Section

### Cell Culture and Maintenance

Hepatic fibroblast derived iPS line ATCC‐HYS0103 Human Induced Pluripotent Stem (IPS) Cells were purchased directly from the vendor. hESC‐Qualified Matrigel (Corning 354277) was used coat culture dishes for feeder‐free expansion of induced pluripotent stem cells and were routinely cultured and maintained in mTeSR1 (STEMCELL Technologies, 85850) in a humidified incubator at 37 °C with 5% CO_2_. Cells were passaged once a week using selective dissociation reagent ReLeSR (STEMCELL Technologies 05872) and seeded using the cell aggregate counting method described in “Plating Human ES and iPS Cells Using the Cell Aggregate Count Method” (Appendix 1) from the STEMCELL Technologies—“Maintenance of Human Pluripotent Stem Cells in mTeSR1” technical handbook to assess the size of aggregates and seed them in low, medium or high densities, as described. All cryopreservation of cells was performed in CryoStor CS10 (STEMCELL Technologies 07930) freezing media.

For the glass controls in the experiments—sterile rh‐Vitronectin (Gibco, Life Technologies, A14700) was used to coat glass cover slips. In case of all experiments, cells were dissociated into a single cell suspension using StemPro Accutase (Gibco A1110501) cell dissociation reagent to facilitate cell counting. In case of single cell dissociation, cells were always seeded with 10 µm Rock inhibitor Y27632 (ATCC ACS‐3030).

### Preparation of PA Coated Cover Slips

Hydrogel based substrates were prepared using chemically modified polyacrylamide and soft lithography. Round glass cover slips (18 mm Diameter) were sonicated and individually placed in a 12‐well tissue culture polystyrene plate, treated with 0.5% 3‐Aminopropyl triethoxysilane (APTS) (Sigma Aldrich A3648) for 3 min then with 0.5% Glutaraldehyde (Sigma Aldrich G6257) for 30 min. The cover slips were thoroughly air dried with the treated surface up. For the polyacrylamide hydrogel coating, 40% solution of Acrylamide (Sigma Aldrich A3553) and 2% solution of Bisacrylamide (Sigma Aldrich 146072) were prepared in distilled water. Solutions pertaining to various elastic modulus were prepared as described in the table.^[^ ^72^ ^]^ The stiffness solution was sandwiched between a hydrophobic glass slide and the treated cover slip. 10% Ammonium Persulfate (APS) and Tetramethylethylenediamine (TEMED) (Sigma Aldrich, 1.10732) were used for polymerization in a covered, moist environment. Cover slips were carefully picked up then treated with hydrazine hydrate 100% (Acros organics 196715000) for up to 1 h to convert amide groups in polyacrylamide to reactive hydrazide groups and then 1 h incubation in a 5% solution of Glacial acetic acid is done before patterning. DI washes performed between each chemical treatment.

### Microcontact Patterning of Polyacrylamide Coated Cover Slips

For microcontact patterning, polydimethylsiloxane (PDMS, Polysciences, Inc.) stamps for 250 and 500 µm diameter circles were prepared by polymerization upon a patterned master of photoresist (SU‐8, MicroChem) created using UV photolithography using a laser printed mask. Sodium periodate (Univar 695‐100G) solution was used to yield free aldehydes in the proteins used for micropatterning. For assisting adhesion of iPS cells, recombinant human Vitronectin (Gibco A14700) was used at a final concentration of 25 ug mL^−1^. The solution was applied to the patterned or non‐patterned PDMS stamps for 30 min, air dried and then applied to the air‐dried PA surface. The stamps were removed after 15 s and the patterned cover slips were sterilized by transferring to a sterile 12‐well TCP dish inside BSCII and 3× Sterile DPBS wash, followed by 12 min of UV exposure and then stored in 4 °C soaked in a 2% Penicillin/Streptomycin + DPBS solution for minimum 6 h. The soaking solution to be removed and warm expansion media to be added to the cover slips before seeding the cells.

### Microcontact Patterning of Glass Cover Slips

The same PDMS stamps were used to assist with physical adsorption of protein on clean, dry glass cover slips. The glass cover slips were sonicated in 100% ethanol and cleaned in a plasma cleaner (PlasmaFlo, Harrick Plasma) to remove all residue. Vitronectin was dissolved in PBS at the final concentration of 25 µg mL^−1^ and applied on clean stamps 30 min/37 °C. The stamps were rinsed, dried, and applied on clean cover slips for 5–7 min allowing for protein adsorption on defined islands. The patterned glass cover slips were sterilized before cell culture and used within 24 h of preparation.

### Cell Seeding on the Micropatterned Cover Slips

The cell culture dishes were monitored for confluency before starting the experiment. All experiments were performed using in mTeSR1 (STEMCELL Technologies, 85850). The cells were dissociated using warm StemPro Accutase (Gibco A1110501) cell dissociation reagent for 6–7 min. Cells were counted using a hemocytometer and seeded in mTeSR1 (STEMCELL Technologies, 85850) and a density of 5 × 10^5^ cells mL^−1^ as a 1 mL per well solution in a 12‐well culture dish with 10 µm Rock inhibitor Y27632 (ATCC ACS‐3030). The medium was replaced with media without Y‐27632 at 24 h for 10 and 100 kPa substrates, and 5 µm Y‐27632 in case of 1 kPa substrates (This was completely removed at 36 h). The cells were allowed to grow on the 4 substrate groups for 48 h.

For BMP4 induction experiments, same seeding method was used, fresh media supplemented with rhBMP4 (STEMCELL Technologies, 78211) at a final concentration of 50 ng mL^−1^ on the 4 substrate groups for 48 h.

For small molecule and protein inhibitor treatments— cell cultures were supplemented with the following concentration of each agent at 6 h of seeding—CHIR99021 (3 µm), SB431542 (5 µm), IWP‐2 (2.5 µm), DKK1 (500 ng mL^−1^), sFRP1 (5 µg mL^−1^), Peptide17 (100 nm).

### Immunocytochemistry—Fixation and Staining

Cell media was replaced with 4% solution of Pierce 16% Formaldehyde (w/v), Methanol‐free (Thermo Fisher Scientific, 2890) diluted in 1X PBS, for 30 min at RT. All washes were performed using Gibco DMEM, no phenol red (Gibco, 31053028). This was done because any contact with PBS would readily detach all colonies from the patterned PA surfaces. 0.1% solution of Triton X‐100 in PBS (v/v) and 3% Bovine Serum Albumin solution (Sigma Aldrich, A3803) in PBS (w/v) was used for permeabilization and blocking. Primary incubation 4 °C overnight/secondary incubation for 1 h at RT in dark. Coverslips were mounted using ProLong Gold Antifade Mountant (Thermo Fisher Scientific, P36930) which contains a DAPI stain.

### 3D Spheroid Culture and Immunostaining

At 48 h, the media was removed, and PBS washes were performed 2–3 times, whilst collecting the PBS after each wash in a separate labeled tube for each condition. As mentioned above, PBS washes would readily dissociate all colonies, but the spheroid structure of the colonies were maintained. 50 µL of warm mTeSR1 was added to a 96‐well Low adherence U‐bottom dish. Individual spheroids were picked up and deposited in the 96 well. After checking each well for only one spheroid each, the wells were topped up with 100 µL of media. The spheroids were grown for 14 days, with media change done every other day. Similarly, spheroids were deposited in the biomaterials and cultured for 7 days. 5000–15000 cells deposited in each well with Y27632 supplemented for controls, cultured 2 days before being deposited in the biomaterials.

For fixation, a cut 200 µL tip was used to pick up the spheroid and transferred to a glass‐bottom 96 well plate and fixed with 4% PFA (Thermo Fisher Scientific, 2890) for 24 h. 2× PBS washes of 1 h each was done followed by 0.2% solution of Triton X‐100 for 2 h and blocking for 1 h. Primary and secondary antibody + Hoescht staining was performed for 24 h each on a slow rocker at RT with 2 × 1 h PBS washes in between.

### In Vivo Teratoma Analysis

The intact spheroids were collected off the 10 kPa PA gels via PBS washes as described above. The spheroids were allowed to settle in the PBS for 10 min at RT, supernatant was removed, and the spheroids were resuspended in 100 µL Matrigel‐GFR (Corning 354230). ATCC hiPSCs and H9 hESCs were used as controls. This was injected in immunocompromised SCID mice subcutaneously and was observed for growth for the next 7 weeks. The teratomas were collected in ice‐cold PBS, fixed in 10% neutral‐buffered formalin solution for 3 days, and transferred in 70% ethanol for 3 days. Paraffin embedded sectioning, mounting and hematoxylin/eosin staining was performed for all sections. Imaging of slides was done using Leica Aperio XT slide scanner. Histology scoring was plotted using Graphpad Prism. All animal experiments were approved by the UNSW Sydney Animal Care and Ethics Committee‐ approval number #18/122B.

### RNA Isolation and qRT‐PCR

For RNA isolation, cells on all four groups of substrates were dissociated using StemPro Accutase (Gibco A1110501) for 5 min. Cell pellets were washed with RT PBS and then with chilled PBS while being centrifuged at 4 °C. All existing PBS was removed, cell pellets were snap‐frozen using liquid nitrogen and stored in −80. RNA isolation was performed using RNeasy Mini Kit (Qiagen 74104) as per kit instructions. cDNA prep was performed using RT2 First Strand Kit (Qiagen 330401) as per kit instructions. The cDNA was added to RT^2^ SYBR Green ROX qPCR Mastermix (Qiagen 330520) and the solution was added to a Custom RT2 PCR Array 384‐well plate and qPCR program was run using QuantStudio 7 Flex Real‐Time PCR System (Applied Biosystems 4485701). Data analysis was performed on https://geneglobe.qiagen.com/us/analyze. MS Excel was used for data compilation and normalization. Heatmaps prepared on GraphPad Prism and Morpheus.

### Confocal Imaging and Data Quantification

The fluorescence imaging was performed on a Zeiss LSM780 or a Zeiss LSM800 confocal microscope, 10×/0.45; 20×/0.8 or 63×/1.4 objectives and acquired using the Zen Black (LSM780) and Zen Blue (LSM800) imaging software (Zeiss). Imaris software (Bitplane) was used for surface rendering of acquired *z*‐stacks (9–12 µm thickness).

Image analysis was performed using FIJI (Fiji is just ImageJ) software, and extra plugins were downloaded when required. Nuclear characteristics analysis was performed using CellProfiler Cell image analysis software from Broad Institute. Characterization of intracellular Yap localizations and nuclear morphology was performed with a customized MATLAB (Mathworks) based GUI (graphic user interface) (available upon request). Data sets were compiled in Microsoft Excel and graphs preparation and statistical analysis was done using GraphPad Prism.

## Conflict of Interest

The authors declare no conflict of interest.

## Author Contributions

S.R. and C.K. contributed equally to this work. P.S., J.P., and K.K. developed the ideas. P.S., S.R., C.K., J.I., T.M., S.N., K.L., P.J., A.Y., E.P., and V.C. performed the experiments and analyzed the data. All authors contributed to writing the manuscript.

## Supporting information

Supporting Information

## Data Availability

The data that support the findings of this study are available from the corresponding author upon reasonable request.

## References

[1] P. P. L. Tam , R. R. Behringer , Mech. Dev. 1997,68, 3. 9431800 10.1016/s0925-4773(97)00123-8

[2] Y. Nakaya , G. Sheng , Dev., Growth Differ. 2008, 50, 755,. 19046163 10.1111/j.1440-169X.2008.01070.x

[3] P. P. Tam , R. S. Beddington , *Ciba Found. Symp*. 1992, 165, 27, discussion 42‐29. 10.1002/9780470514221.ch3 1516473

[4] P. Gadue , T. L. Huber , P. J. Paddison , G. M. Keller , Proc. Natl. Acad. Sci. U. S. A. 2006, 103, 16806. 17077151 10.1073/pnas.0603916103 PMC1636536

[5] J. W.h Wen , R. Winklbauer , eLife 2017, 6, e27190. 28826499 10.7554/eLife.27190 PMC5589415

[6] S. Pfister , K. A. Steiner , P. P. L. Tam , Gene Expression Patterns 2007, 7, 558. 17331809 10.1016/j.modgep.2007.01.005

[7] J. A. Rivera‐Pérez , T. Magnuson , Dev. Biol. 2005, 288, 363. 16289026 10.1016/j.ydbio.2005.09.012

[8] R. Hiramatsu , T. Matsuoka , C. Kimura‐Yoshida , S.‐W. Han , K. Mochida , T. Adachi , S. Takayama , I. Matsuo , Dev. Cell 2013, 27, 131. 24176640 10.1016/j.devcel.2013.09.026

[9] M. F. Pera , Development 2017, 144, 1923. 28559237 10.1242/dev.151191

[10] I. Roszko , A. Sawada , L. Solnica‐Krezel , Semin. Cell Dev. Biol. 2009, 20, 986,. 19761865 10.1016/j.semcdb.2009.09.004 PMC2796982

[11] (!!! INVALID CITATION !!! 9,10).

[12] A. Warmflash , B. Sorre , F. Etoc , E. D. Siggia , A. H. Brivanlou , Nat. Methods 2014, 11, 847. 24973948 10.1038/nmeth.3016 PMC4341966

[13] A. Deglincerti , et al., Nat. Protoc. 2016, 11, 2223,. 27735934 10.1038/nprot.2016.131 PMC5821517

[14] Z. Zhang , S. Zwick , E. Loew , J. S. Grimley , S. Ramanathan , bioRxiv 2018, 491290.

[15] K. Pfister , D. R. Shook , C. Chang , R. Keller , P. Skoglund , Development 2016, 143, 715. 26884399 10.1242/dev.128090 PMC4760319

[16] J. S. Morales , J. Raspopovic , L. Marcon , Stem Cell Rep. 2021, 16, 1039. 10.1016/j.stemcr.2021.03.026 PMC8185431 33979592

[17] Q. Smith , E. Stukalin , S. Kusuma , S. Gerecht , S. X. Sun , Sci. Rep. 2015, 5, 12617. 26227093 10.1038/srep12617 PMC4521170

[18] J. Muncie , N. Ayad , J. Lakins , V. Weaver , Dev. Cell 2020, 55, 679. 33207224 10.1016/j.devcel.2020.10.015 PMC7755684

[19] J. Lee , A. A. Abdeen , D. Zhang , K. A. Kilian , Biomaterials 2013, 34, 8140. 23932245 10.1016/j.biomaterials.2013.07.074

[20] J. Swift , I. L. Ivanovska , A. Buxboim , T. Harada , P. C. D. P. Dingal , J. Pinter , J. D. Pajerowski , K. R. Spinler , J.‐W. Shin , M. Tewari , F. Rehfeldt , D. W. Speicher , D. E. Discher , Science 2013, 341, 1240104. 23990565 10.1126/science.1240104 PMC3976548

[21] P. Wang , R. T. Rodriguez , J. Wang , A. Ghodasara , S. K. Kim , Cell Stem Cell 2011, 8, 335. 21362573 10.1016/j.stem.2011.01.017 PMC3063711

[22] Z. Ma , et al., Nat. Commun. 2015, 6, 7413,. 26172574 10.1038/ncomms8413 PMC4503387

[23] Y.‐F. Chen , Y.‐S. J. Li , C.‐H. Chou , M. Y. Chiew , H.‐D. Huang , J. H.‐C. Ho , S. Chien , O. K. Lee , Sci. Adv. 2020, 6, 0264. 10.1126/sciadv.aay0264 PMC7002135 32076643

[24] M. Jaramillo , S. S. Singh , S. Velankar , P. N. Kumta , I. Banerjee , J. Tissue Eng. Regener. Med. 2015, 9, 14. 10.1002/term.1602 PMC4130804 23008262

[25] I. Martyn , T. Y. Kanno , A. Ruzo , E. D. Siggia , A. H. Brivanlou , Nature 2018, 558, 132. 29795348 10.1038/s41586-018-0150-y PMC6077985

[26] A. Abedini , C. Sayed , L. E. Carter , D. Boerboom , B. C. Vanderhyden , Sci. Rep. 2020, 10, 9695. 32546756 10.1038/s41598-020-66559-9 PMC7298016

[27] C. E. Ford , G. Punnia‐Moorthy , C. E. Henry , E. Llamosas , S. Nixdorf , J. Olivier , R. Caduff , R. L. Ward , V. Heinzelmann‐Schwarz , Gynecol. Oncol. 2014, 134, 338. 24924122 10.1016/j.ygyno.2014.06.004

[28] S.‐W. Cha , E. Tadjuidje , Q. Tao , C. Wylie , J. Heasman , Development 2008, 135, 3719. 18927149 10.1242/dev.029025

[29] K. M. Hardy , R. J. Garriock , T. A. Yatskievych , S. L. D'agostino , P. B. Antin , P. A. Krieg , Dev. Biol. 2008, 320, 391. 18602094 10.1016/j.ydbio.2008.05.546 PMC2539108

[30] K. A. Kilian , B. Bugarija , B. T. Lahn , M. Mrksich , Proc. Natl. Acad. Sci. U. S. A. 2010, 107, 4872. 20194780 10.1073/pnas.0903269107 PMC2841932

[31] S. Nemec , J. Lam , J. Zhong , C. Heu , P. Timpson , Q. Li , J. Youkhana , G. Sharbeen , P. A. Phillips , K. A. Kilian , Adv. Biol. 2021, 5, 2000525. 10.1002/adbi.202000525 33754491

[32] J. Lim , J. P. Thiery , Development 2012, 139, 3471. 22949611 10.1242/dev.071209

[33] V. Halacheva , M. Fuchs , J. Dönitz , T. Reupke , B. Püschel , C. Viebahn , Dev. Dyn. 2011, 240, 1905. 21761476 10.1002/dvdy.22687

[34] M. F. Basilicata , M. Frank , D. Solter , T. Brabletz , M. P. Stemmler , Sci. Rep. 2016, 6, 26562. 27217206 10.1038/srep26562 PMC4877576

[35] A. Cano , M. A. Pérez‐Moreno , I. Rodrigo , A. Locascio , M.­A. J. Blanco , M. G. Del Barrio , F. Portillo , M. A. Nieto , Nat. Cell Biol. 2000, 2, 76. 10655586 10.1038/35000025

[36] J. D. Mih , A. Marinkovic , F. Liu , A. S. Sharif , D. J. Tschumperlin , J. Cell Sci. 2012, 125, 5974. 23097048 10.1242/jcs.108886 PMC3585515

[37] J. K. Virdi , P. Pethe , Tissue Eng. Regener. Med. 2021, 18, 199. 10.1007/s13770-020-00301-4 PMC8012461 33230800

[38] H.‐T. Hsu , C. Estarás , L. Huang , K. A. Jones , Stem Cell Rep. 2018, 11, 1357. 10.1016/j.stemcr.2018.10.013 PMC6294113 30449705

[39] E. Stronati , S. Giraldez , L. Huang , E. Abraham , G. R. Mcguire , H.‐T. Hsu , K. A. Jones , C. Estarás , Stem Cell Rep. 2022, 17, 211. 10.1016/j.stemcr.2021.12.012 PMC8828531 35063126

[40] K. Molugu , T. Harkness , J. Carlson‐Stevermer , R. Prestil , N. J. Piscopo , S. K. Seymour , G. T. Knight , R. S. Ashton , K. Saha , Biophys. J. 2020, 118, 2086. 31699335 10.1016/j.bpj.2019.10.014 PMC7203070

[41] M. A. Garcia , R. Rickman , J. Sero , Y. Yuan , C. Bakal , bioRxiv 2019, 689737, 10.1101/689737.

[42] O. Voiculescu , F. Bertocchini , L. Wolpert , R. E. Keller , C. D. Stern , Nature 2007, 449, 1049. 17928866 10.1038/nature06211

[43] E. Rozbicki , M. Chuai , A. I. Karjalainen , F. Song , H. M. Sang , R. Martin , H.‐J. Knölker , M. P. Macdonald , C. J. Weijer , Nat. Cell Biol. 2015, 17, 397. 25812521 10.1038/ncb3138 PMC4886837

[44] M. Tada , M. L. Concha , C.‐P. Heisenberg , Semin. Cell Dev. Biol. 2002, 13, 251,. 12137734 10.1016/s1084-9521(02)00052-6

[45] I. Lian , et al., Genes Dev. 2010, 24, 1106,. 20516196 10.1101/gad.1903310 PMC2878649

[46] Z. Zhang , Z. Lin , Z. Zhou , H. C. Shen , S. F. Yan , A. V. Mayweg , Z. Xu , N. Qin , J. C. Wong , Z. Zhang , Y. Rong , D. C. Fry , T. Hu , ACS Med. Chem. Lett. 2014, 5, 993. 25221655 10.1021/ml500160m PMC4160762

[47] I. Martyn , A. H. Brivanlou , E. D. Siggia , Development 2019, 146, 172791. 10.1242/dev.172791 PMC6451321 30814117

[48] S. Vianello , M. P. Lutolf , Dev. Cell 2019, 48, 751. 30913407 10.1016/j.devcel.2019.02.024

[49] B. Guillaume , D. Wisniewski , C. Picart , M. Thery , M. Puceat , S. Lowell , Development 2018, 145, 166025. 10.1242/dev.166025 PMC6176930 30115626

[50] X. Xue , Y. Sun , A. M. Resto‐Irizarry , Y. Yuan , K. M. Aw Yong , Y. Zheng , S. Weng , Y. Shao , Y. Chai , L. Studer , J. Fu , Nat. Mater. 2018, 17, 633. 29784997 10.1038/s41563-018-0082-9 PMC6622450

[51] G. Sahni , S. Y. Chang , J. T. C. Meng , J. Z. Y. Tan , J. J. C. Fatien , C. Bonnard , K. H. Utami , P. W. Chan , T. T. Tan , U. Altunoglu , H. Kayserili , M. Pouladi , B. Reversade , Y. C. Toh , Adv. Sci. 2021, 8, 2001100. 10.1002/advs.202001100 PMC7927627 33717833

[52] Q. Smith , N. Rochman , A. M. Carmo , D. Vig , X. Y.i Chan , S. Sun , S. Gerecht , Proc. Natl. Acad. Sci. U. S. A. 2018, 115, 8167. 30038020 10.1073/pnas.1808021115 PMC6094121

[53] S. Pagliari , et al., Cell Death Differ. 2021, 28, 1193,. 33116297 10.1038/s41418-020-00643-5 PMC8027678

[54] M. Pekkanen‐Mattila , M. Pelto‐Huikko , V. Kujala , R. Suuronen , H. Skottman , K. Aalto‐Setälä , E. Kerkelä , Histochem. Cell Biol. 2010, 133, 595. 20369364 10.1007/s00418-010-0689-7

[55] K. M. Loh , B. Lim , Cell Stem Cell 2011, 8, 363. 21474100 10.1016/j.stem.2011.03.013

[56] I. Aksoy , R. Jauch , J. Chen , M. Dyla , U. Divakar , G. K. Bogu , R. Teo , C. K. Leng Ng , W. Herath , S. Lili , A. P. Hutchins , P. Robson , P. R. Kolatkar , L. W. Stanton , EMBO J. 2013, 32, 938. 23474895 10.1038/emboj.2013.31 PMC3616284

[57] Y. Lin , X.‐Y. Li , A. L. Willis , C. Liu , G. Chen , S. J. Weiss , Nat. Commun. 2014, 5, 3070. 24401905 10.1038/ncomms4070 PMC4115678

[58] S. A. Morris , R. T. Y. Teo , H. Li , P. Robson , D. M. Glover , M. Zernicka‐Goetz , Proc. Natl. Acad. Sci. U. S. A. 2010, 107, 6364. 20308546 10.1073/pnas.0915063107 PMC2852013

[59] C. A. Séguin , J. S. Draper , A. Nagy , J. Rossant , Cell Stem Cell 2008, 3, 182. 18682240 10.1016/j.stem.2008.06.018

[60] K. K. Niakan , H. Ji , R. Maehr , S. A. Vokes , K. T. Rodolfa , R. I. Sherwood , M. Yamaki , J. T. Dimos , A. E. Chen , D. A. Melton , A. P. Mcmahon , K. Eggan , Genes Dev. 2010, 24, 312. 20123909 10.1101/gad.1833510 PMC2811832

[61] R. S. P. Beddington , P. Rashbass , V. Wilson , Development 1992, 116, 157. 1299362

[62] T. Faial , A. S. Bernardo , S. Mendjan , E. Diamanti , D. Ortmann , G. E. Gentsch , V. L. Mascetti , M. W. B. Trotter , J. C. Smith , R. A. Pedersen , Development 2015, 142, 2121. 26015544 10.1242/dev.117838 PMC4483767

[63] Q. Chen , Y. Zhang , D. Elad , A. J. Jaffa , Y. Cao , X. Ye , E. Duan , Mol. Aspects Med. 2013, 34, 1024. 22921800 10.1016/j.mam.2012.07.017

[64] M. Williams , C. Burdsal , A. Periasamy , M. Lewandoski , A. Sutherland , Dev. Dyn. 2012, 241, 270. 22170865 10.1002/dvdy.23711 PMC3266444

[65] G. Cui , et al., Dev., Growth Differ. 2018, 60, 463,. 30368783 10.1111/dgd.12568

[66] N. Gjorevski , E. Boghaert , C. M. Nelson , Cancer Microenviron. 2012, 5, 29. 21748438 10.1007/s12307-011-0076-5 PMC3343202

[67] P. Liu , M. Wakamiya , M. J. Shea , U. Albrecht , R. R. Behringer , A. Bradley , Nat. Genet. 1999, 22, 361. 10431240 10.1038/11932

[68] P. Gadue , T. L. Huber , P. J. Paddison , G. M. Keller , Proc. Natl. Acad. Sci. U. S. A. 2006, 103, 16806. 17077151 10.1073/pnas.0603916103 PMC1636536

[69] T. A. Blauwkamp , S. Nigam , R. Ardehali , I. L. Weissman , R. Nusse , Nat. Commun. 2012, 3, 1070. 22990866 10.1038/ncomms2064 PMC3657997

[70] K. M. Loh , L. T. Ang , J. Zhang , V. Kumar , J. Ang , J. Q. Auyeong , K. L. Lee , S. H. Choo , C. Y. Y. Lim , M. Nichane , J. Tan , M. S. Noghabi , L. Azzola , E. S. Ng , J. Durruthy‐Durruthy , V. Sebastiano , L. Poellinger , A. G. Elefanty , E. G. Stanley , Q. Chen , S. Prabhakar , I. L. Weissman , B. Lim , Cell Stem Cell 2014, 14, 237. 24412311 10.1016/j.stem.2013.12.007 PMC4045507

[71] S. Vianello , M. Lutolf , In vitro endoderm emergence and self‐organisation in the absence of extraembryonic tissues and embryonic architecture., 2020.

[72] A. J. Engler , S. Sen , H. L. Sweeney , D. E. Discher , Cell 2006, 126, 677. 16923388 10.1016/j.cell.2006.06.044

