# Supplementary material for: Defined Microenvironments Trigger In Vitro Gastrulation in Human Pluripotent Stem Cells
Source: Adv Sci (Weinh). 2022 Dec 15;10(5):2203614. doi: 10.1002/advs.202203614 (PMC9929265; doi:10.1002/advs.202203614)
Supplement: Supplementary file 1 — Supporting Information [file ADVS-10-2203614-s001.pdf]

Defined microenvironments trigger *in vitro* gastrulation in pluripotent stem cells

Pallavi Srivastava[1,3,4], Sara Romanazzo[1], Chantal Kopecky[1,4], Stephanie Nemec[1,2], Jake Ireland[1], Thomas G. Molley[1,2], Pavithra Jayathilaka[1,2], Elvis Pandzic[5], Avani Yeola[4], Vashe Chandrakanthan[3,4], John Pimanda[3,4], Kristopher Kilian[1,2,4*]

[1]*School of Chemistry, Australian Centre for NanoMedicine, University of New South Wales, Sydney NSW, Australia*

[2]*School of Materials Science and Engineering, University of New South Wales, Sydney NSW, Australia*

[3]*School of Medical Sciences, University of New South Wales, Sydney NSW, Australia*

[4]*Adult Cancer Program, Lowy Cancer Research Centre, UNSW Sydney, Sydney, NSW 2052, Australia*

[5] *Katharina Gaus Light Microscopy Facility, Mark Wainwright Analytical Centre, University of New South Wales, Sydney, NSW 2052*

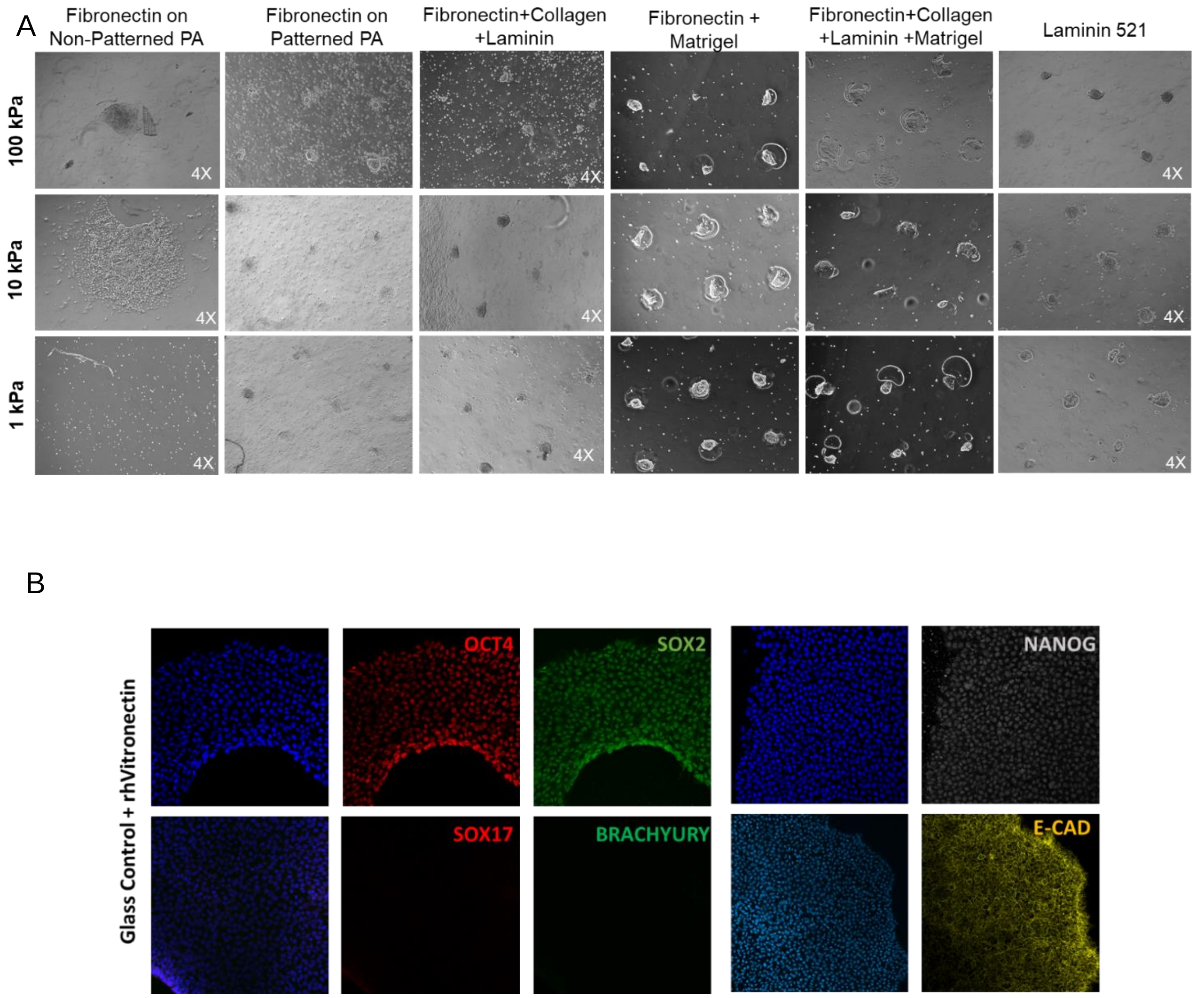

Figure S1: A) The panel of various ECM proteins tested for cell adhesion on polyacrylamide hydrogels, shows inadequate adhesion and proliferation in all combinations at 48 hours.

B) ATCC hiPSC characterisation

Scale bars: 100 µm

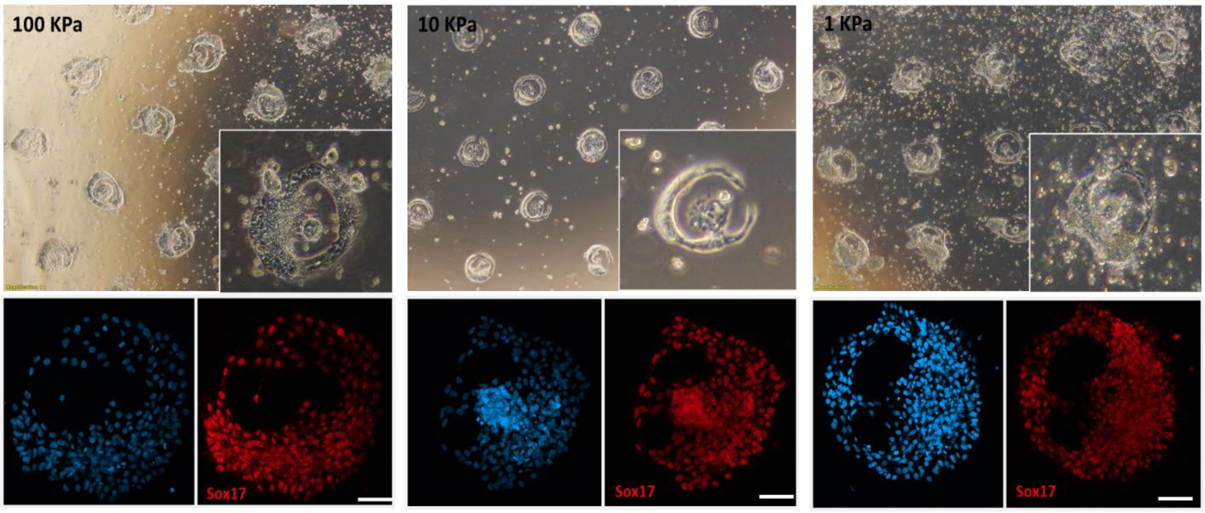

Figure S2: Brightfield and IF images of hiPSCs seeded on PA gel substrates with 250 µm circular patterns at 12 hours. The cells seem to circle the colony boundary before depositing towards the colony centre. The cells were seen to be of Sox17+ endodermal identity as early as 12h post seeding.

Scale bars: 50 µm

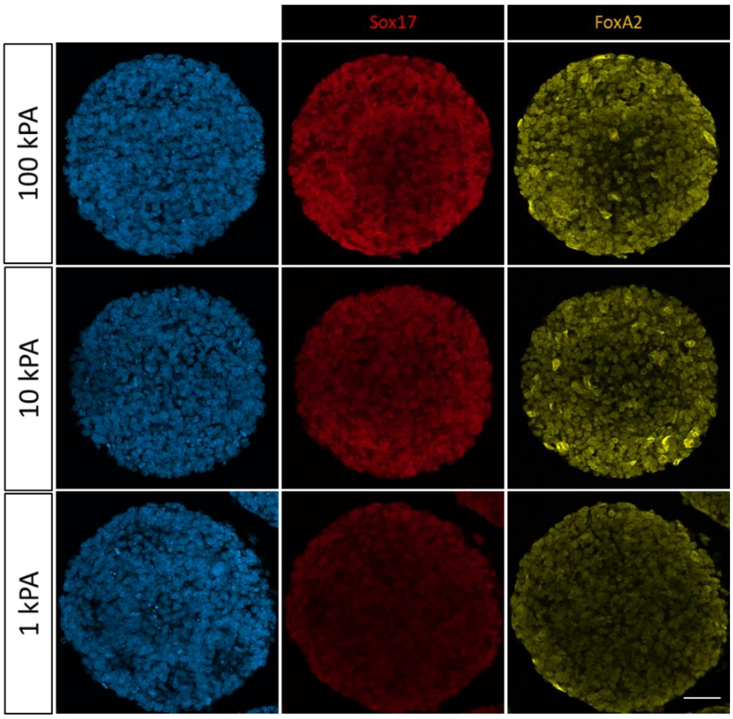

Figure S3 – Colonies 250 µm circles on PA gels positive for Sox17 as well as FoxA2

Scale bar – 50 µm

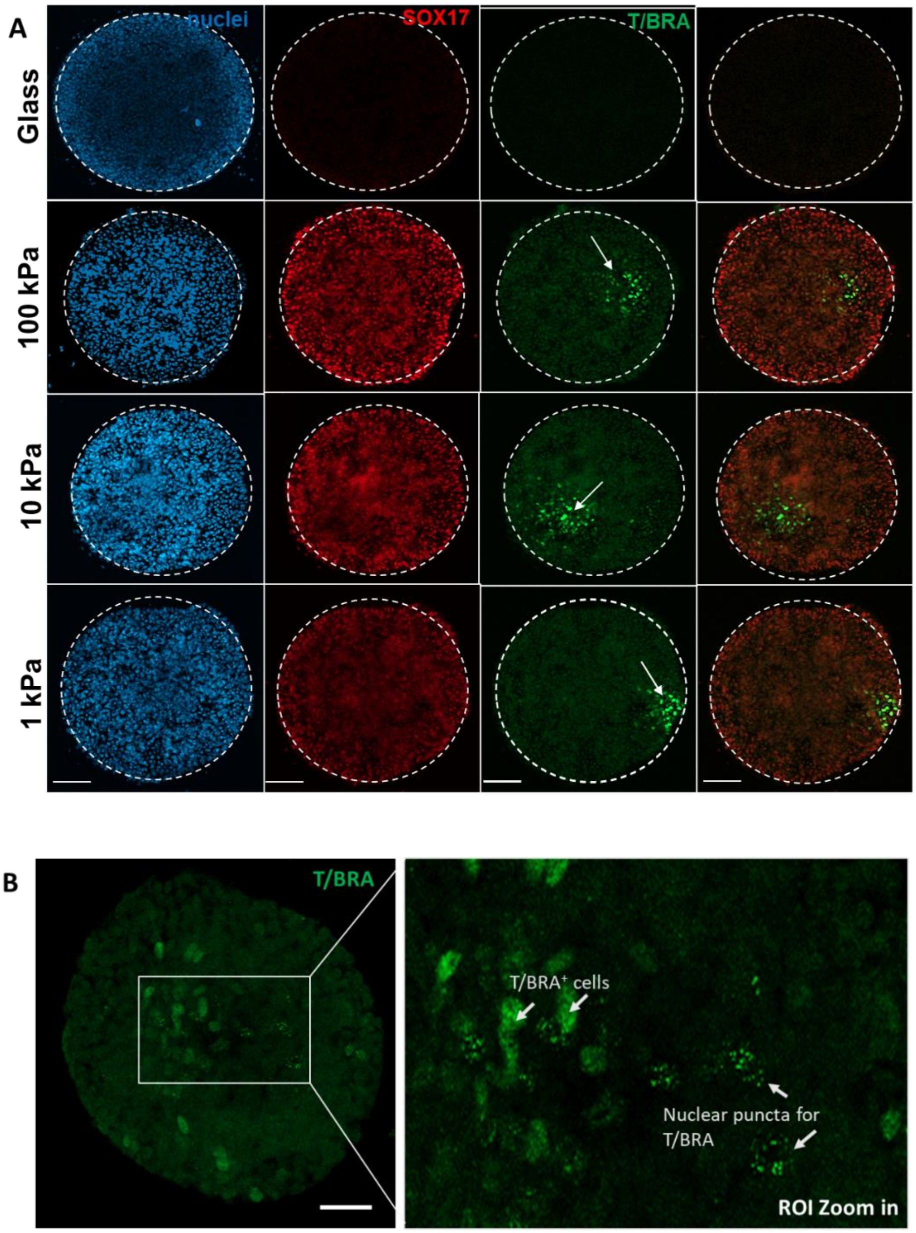

Figure S4 – A) Colonies 500 µm circles on glass and PA gels positive for Sox17 and display a distinct T/BRACHYURY+ cluster within. B) Magnified ROI in a 10 kPa colony centre showing the appearance of both T/BRA+ cells and T/BRA+ puncta within nuclei in the same colony,

Scale Bars: 100 µm

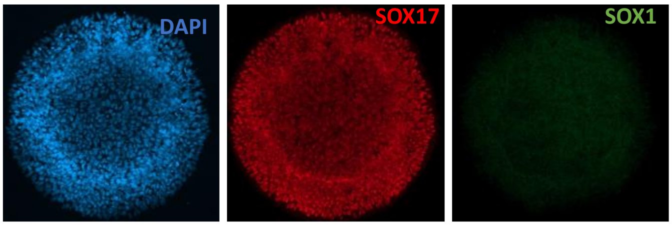

Figure S5 – Colonies on PA gels demonstrating negative staining for ectoderm marker SOX1

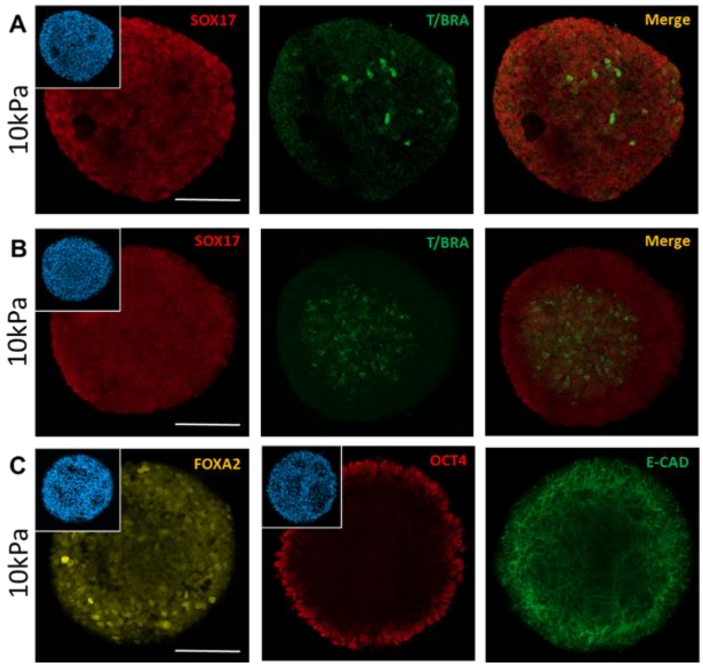

Figure S6: Immunofluorescent images of H9 hESCs seeded on 10 kPa PA gel substrates with 250 µm circular patterns at 48 hours. A) The hESCs assumed an endodermal SOX17+ identity with a mesodermal T/BRACHYURY+ cluster within. B) In absence of a mesodermal cluster, nuclear puncta were observed in colony centres in response to T/BRACHYURY staining. C) Majority of SOX17+ cells co-expressed endodermal marker FOXA2, an OCT4 annulus at the colony edges was observed, along with loss of E-CADHERIN in the colony centres.

Scale bars: 100 µm

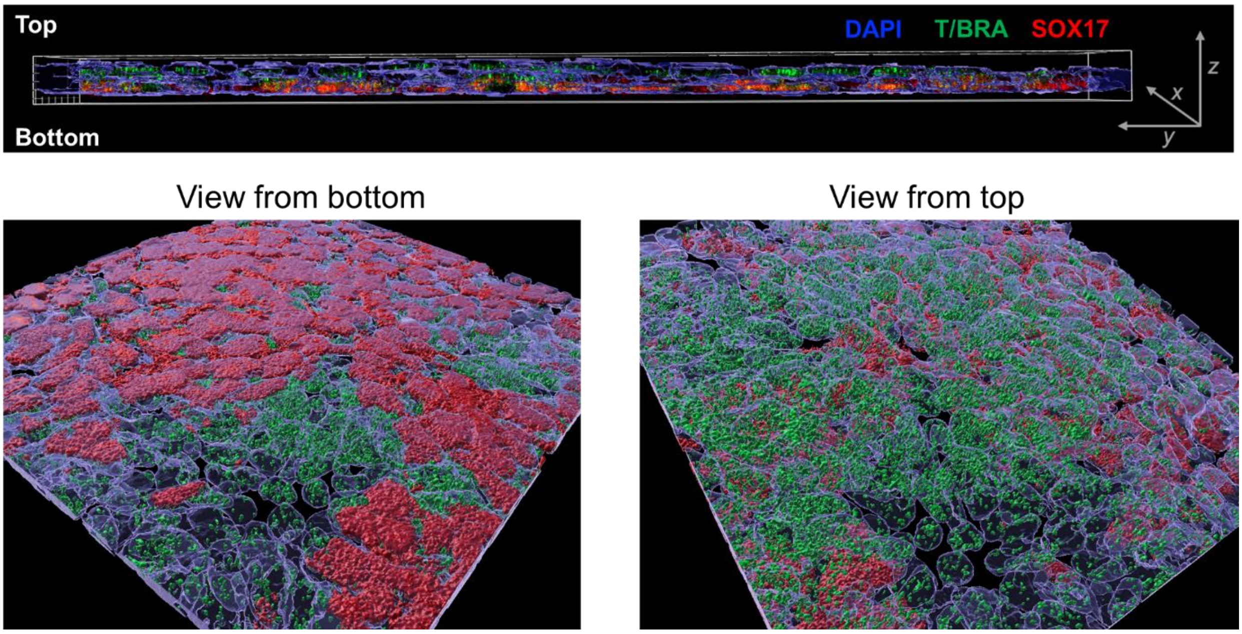

Figure S7: Representative images of Sox 17 and T/Bra expression throughout the multilayered 10 kPa colonies.

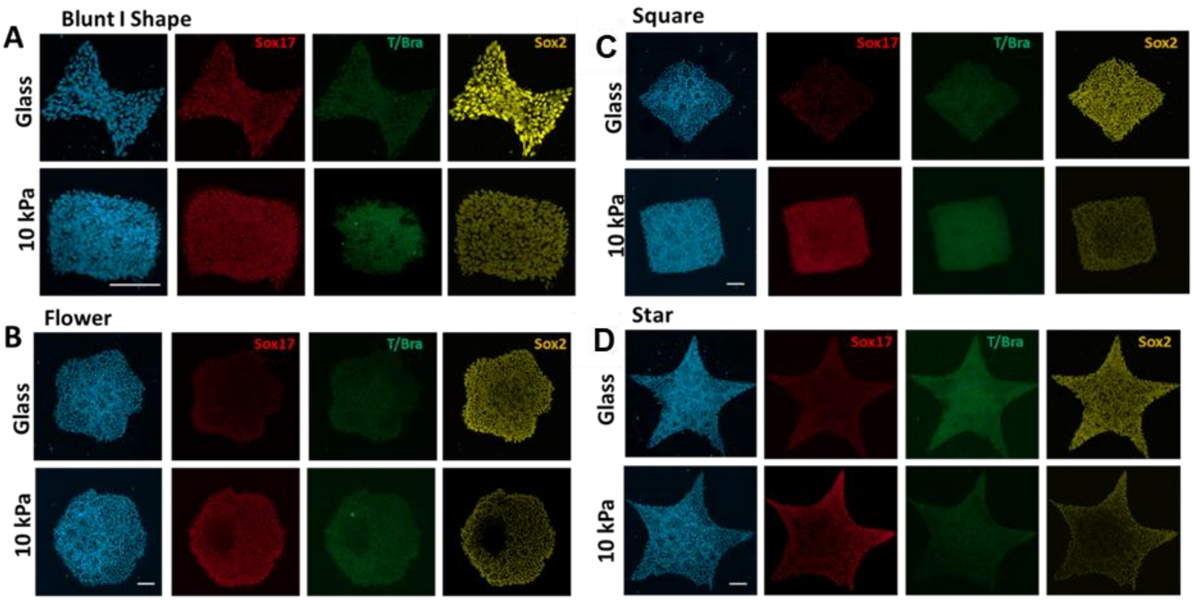

Figure S8: Immunofluorescent images of ATCC hiPSCs seeded on glass 10 kPa PA gel substrates patterned using various shapes at 48 hours. A) Blunt 'I' shape B) Flower C) Sharp 'I' shape D) Square, and E) Star.

Scale bars: 100 µm

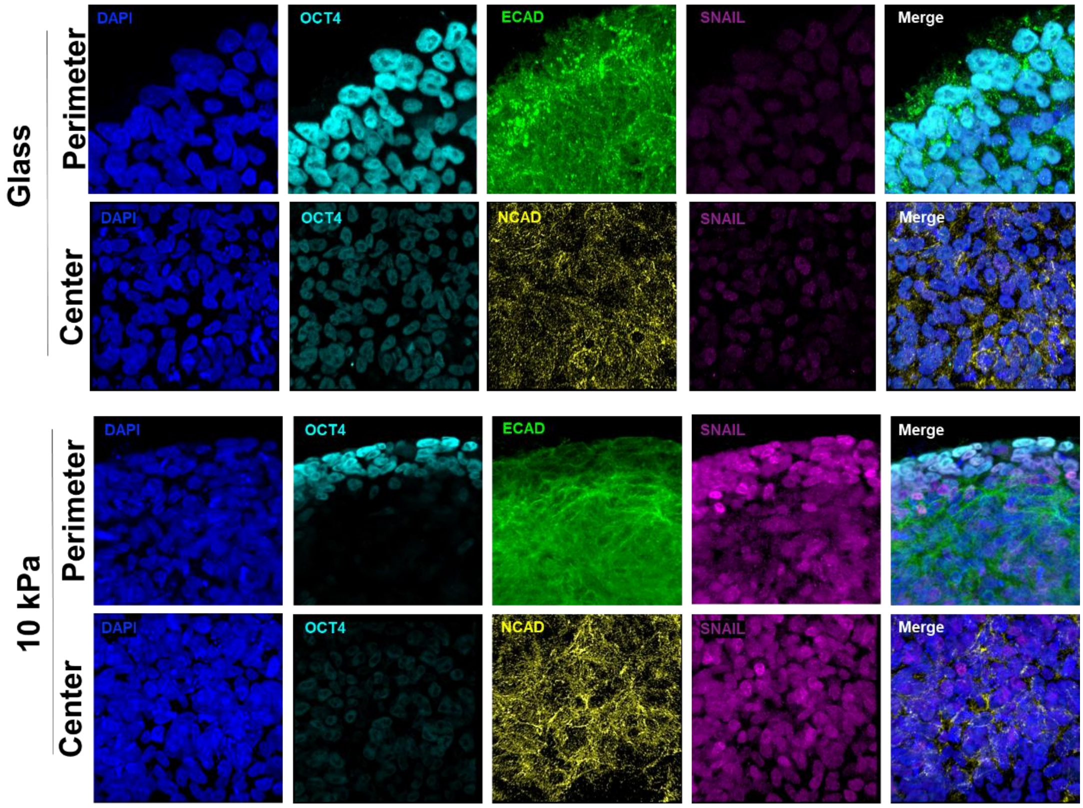

Figure S9: High resolution imaging for markers of pluripotency and EMT in colonies on glass and hydrogel substrates at 48 hours.

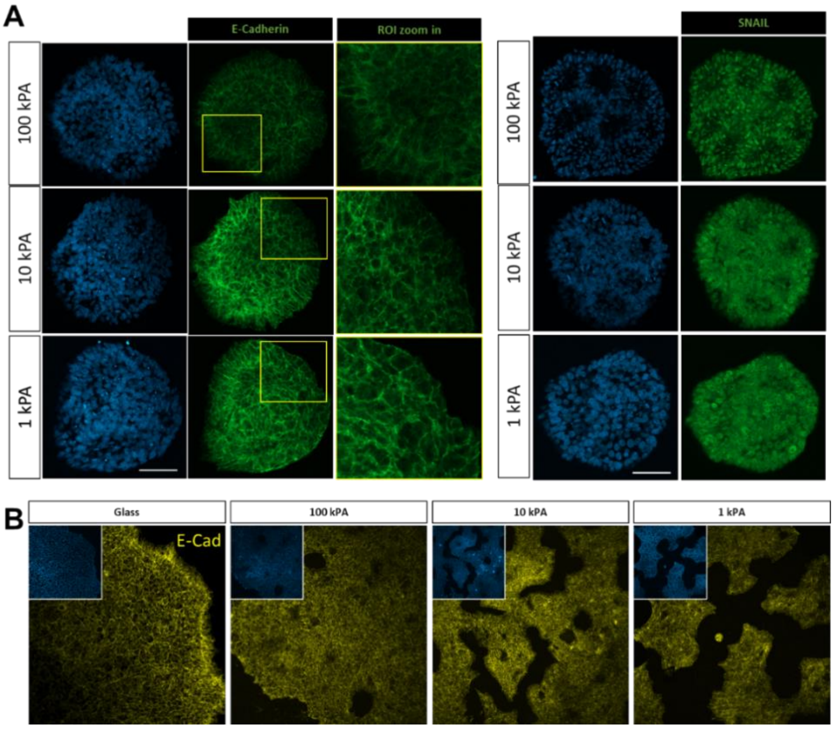

Figure S10: EMT Signifiers on non-BMP4 experiments A) Discontinuous regions of E-Cadherin and Expression of EMT marker snail in the 250um diameter colonies: signifies occurrence of EMT: Significant discontinuity and cytoplasmic localization of E-Cadherin in the colonies on the PA hydrogel substrates. Global Expression of SNAIL on the 3 stiffness substrates, however the clearest expression is observed on 100kPa. Both these act as an important signifier of EMT. B) E-cad expression on glass and Non-Patterned gels

Scale bars: 100 µm

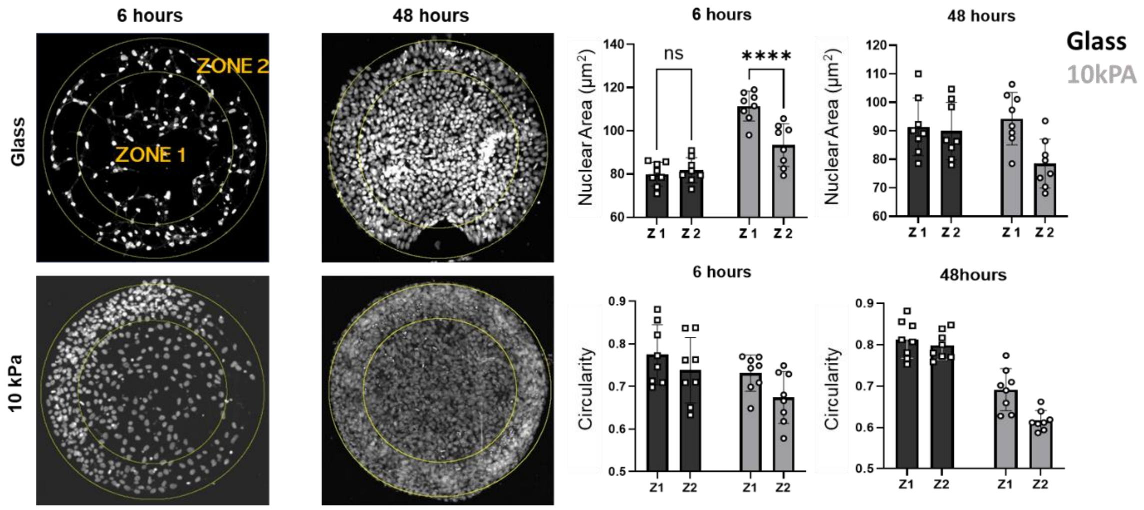

Figure S11: Comparison of nuclear shape characteristics at the edge (ZONE 2) and centre (ZONE 1) of micropatterned islands. ****P<0.05

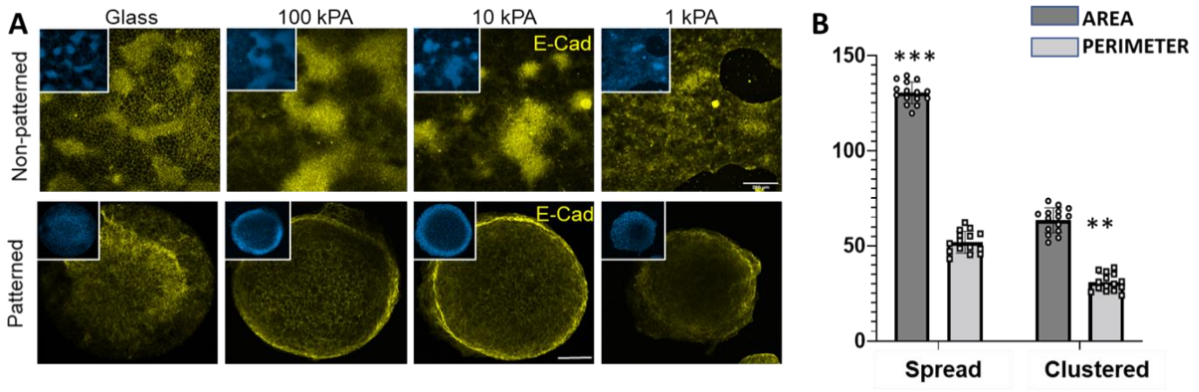

Figure S12: A) E-cad expression pattern on BMP4 treated non–patterned and patterned colonies. B) Nuclear size comparison for spread and clustered cells after 48hr treatment with BMP4 (N=15)

Scale bars: 100 µm

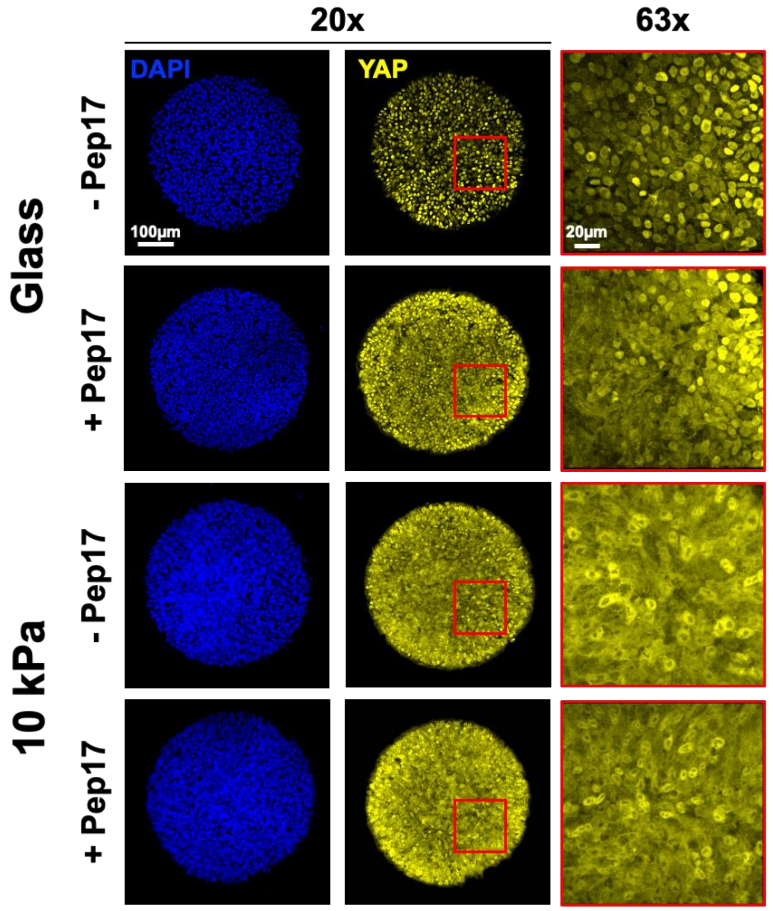

Figure S13: Low (20x) and high magnification (63x) comparison of YAP localisation for iPSCs cultured with and without Pep17

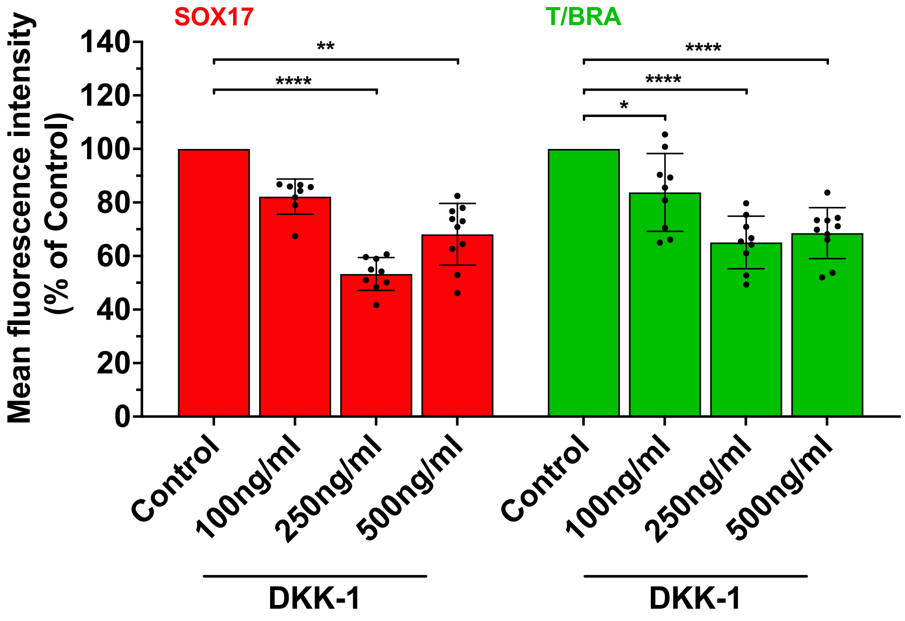

Figure S14: Change in SOX17 and T/BRACHYURY expression upon treatment of different doses of DKK1.

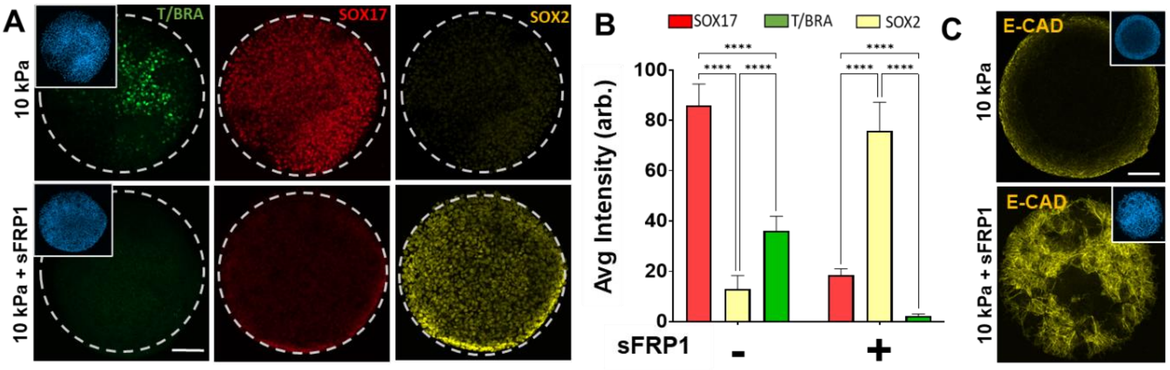

Figure S15: **Total WNT disruption using sFRP1 A)** Immunofluorescence images of micropatterned hiPSCs on 10 kPa hydrogels with and without total WNT inhibition (sFRP1) **B**) corresponding quantitation comparing SOX17 and SOX2 intensity levels after total WNT inhibition on 10 kPa hydrogels (N=10) arb. = Arbitrary units. **** = p<0.0001, ** =p<0.01 (Two-way ANOVA) **C**) E-CAD expression pattern with and without total WNT disruption using sFRP1

Scale Bars: 100 µm

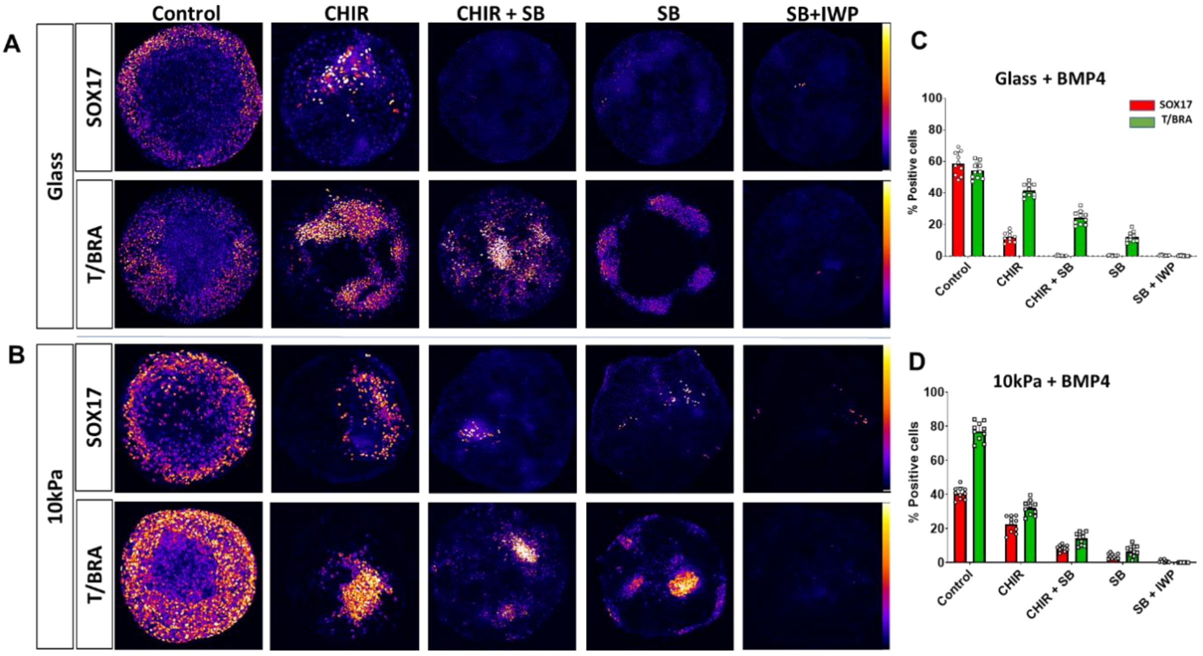

Figure S16: Small molecule inhibition on BMP4 sets: **A)** Heat maps for glass patterns at 48 hours – combinations of small molecule inhibitors for Wnt and Nodal SB = SB431542, IWP2 **B)** Heat maps for 10 kPa patterns at 48 hours – combinations of small molecule inhibitors for Wnt and Nodal **C)** Percent positive quantification for SOX17 and T/BRACHYURY in glass patterns + BMP4 N=10 $p<0.001$ **D)** Percent positive quantification for SOX17 and T/BRACHYURY in 10kPa patterns + BMP4 N=10 $p<0.05$

Scale bars - 100 µM.

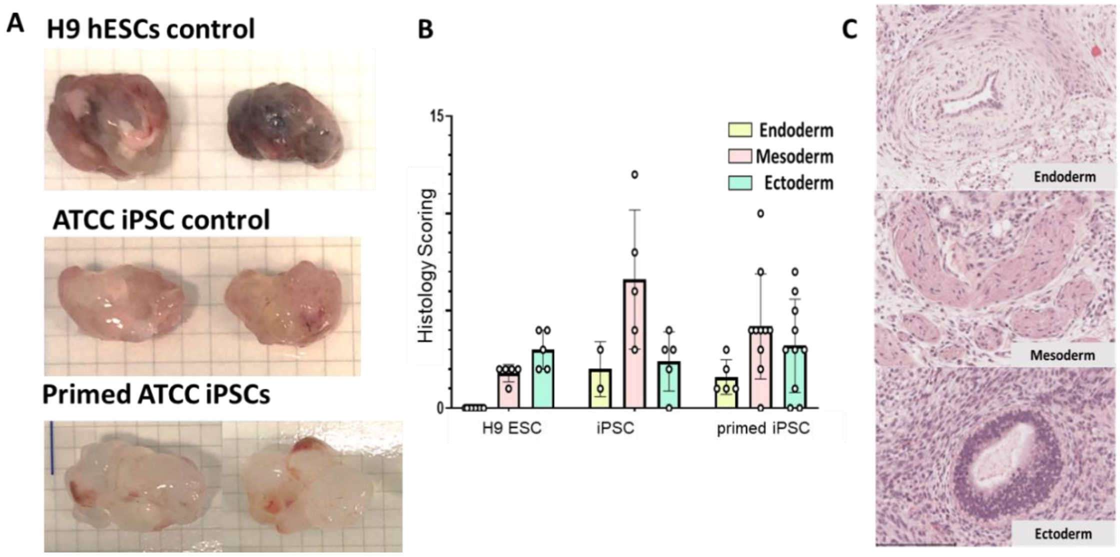

Figure S17: A) Comparative images for excised *in vivo* teratomas from H9 hESCs From ATCC hiPSCs from primed PA spheroids B) histology scoring of germ layer derivatives in all three groups

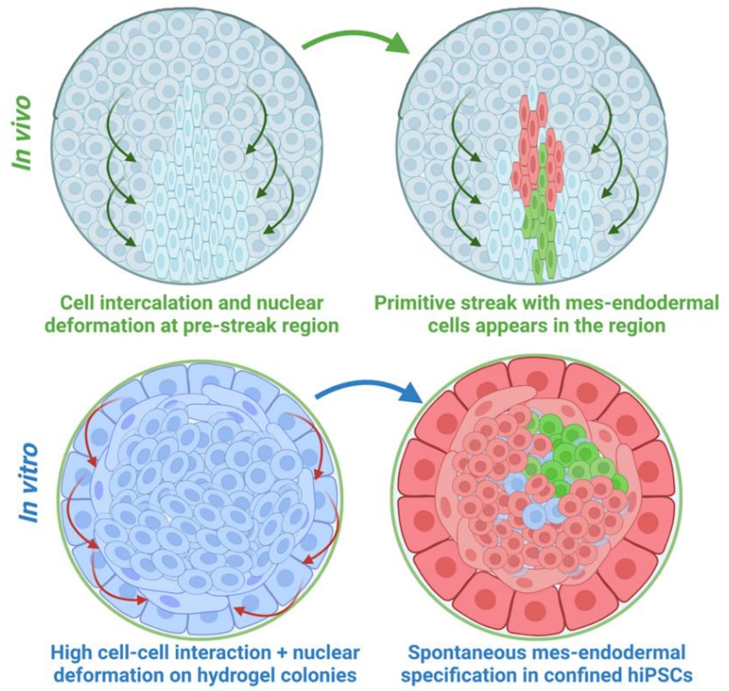

Figure S18: Top: Schematic underlining cellular movements along the posterior epiblast in a gastrulating embryo *in vivo*. The cells in this region undergo higher cell division, higher junctional stress, and nuclear deformation. The first mes-endodermal progenitors appear in the same region, called primitive streak

Bottom: Schematic shows *in vitro* platform reminiscent of the pre-primitive streak region. Higher cell-cell interaction and cell division due to confinement on hydrogel led to nuclear deformation and mes-endodermal specification.

Table 1: <u>Primary Antibodies and their respective dilutions</u>

**Antibody name**	**Cat. No**	**Dilution used**
Oct4	Goat Polyclonal Anti-Oct4 antibody (ab27985)	1:500
Nanog	Nanog Mouse Monoclonal Antibody (hNanog.2), eBioscience 14-5768-82	1:400
Sox17	RDSAF1924 R&D Systems Human SOX17 Affinity Purified Goat Polyclonal Ab	1:300
T/Brachyury	ab209665 Rabbit monoclonal [EPR18113] to Brachyury	1:500
Sox2	ab79351 Mouse monoclonal [9-9-3] to SOX2	1:500
E-Cadherin	Mouse monoclonal [M168] to E Cadherin ab76055	1:500
FoxA2	Mouse monoclonal [7E6] to FOXA2 ab60721	1:500
Sox1	SOX1 Recombinant Rabbit Monoclonal Antibody MA5-32447	1:500
Snail	SNAIL Rabbit Monoclonal Antibody (F.31.8) MA5-14801	1:400
N-Cadherin	N-Cadherin Rabbit Antibody Cell Signalling Technology 4061	1:500
YAP	Anti-YAP1 mouse monoclonal Antibody (63.7): sc-101199	1:400

Table 2: <u>Secondary Antibodies and their respective dilutions</u>

**Antibody name**	**Cat. No**	**Dilution Used**
Anti-goat 647	ab150131 Donkey Anti-Goat IgG H&L (Alexa Fluor® 647)	1:400-1:600
Anti-rabbit 488	Donkey anti-Rabbit IgG (H+L) Highly Cross-Adsorbed Secondary Antibody, Alexa Fluor 488 A-21206	1:400-1:600
Anti-Mouse 555	Goat anti-Mouse IgG (H+L) Highly Cross-Adsorbed Secondary Antibody, Alexa Fluor 555 A-21424	1:400-1:600
Phalloidin 647	Phalloidin–Atto 647N 65906-10NMOL	1:200

Table 3: Gene list for custom designed RT2 PCR Array 384-well plate for quantitative PCR analysis

Symbol	Description
DNMT3B	DNA (cytosine-5-)-methyltransferase 3 beta
GDF3	Growth differentiation factor 3
Lefty1	Left-right determination factor 1
Nanog	Nanog homeobox
PODXL	Podocalyxin-like
POU5F1	POU class 5 homeobox 1
CCDC42	Coiled-coil domain containing 42
FGF5	Fibroblast growth factor 5
foxD3	Forkhead box D3
OTX2	Orthodenticle homeobox 2
ZIC1	Zic family member 1
GBX2	Gastrulation brain homeobox 2
NEUROG2	Neurogenin 2
BMP4	Bone morphogenetic protein 4
SYNE2	Spectrin repeat containing, nuclear envelope 2
EMD	Emerin
GATA2	GATA binding protein 2
HAND1	Heart and neural crest derivatives expressed 1
WDR5	WD repeat domain 5
MIXL1	Mix paired-like homeobox
PDGFRA	Platelet-derived growth factor receptor, alpha polypeptide
RUNX1	Runt-related transcription factor 1
T	T, brachyury homolog (mouse)
FOXA1	Forkhead box A1
HDAC1	Histone deacetylase 1
GATA6	GATA binding protein 6
HNF4A	Hepatocyte nuclear factor 4, alpha
SOX17	SRY (sex determining region Y)-box 17
LMNA	Lamin A/C
FABP7	Fatty acid binding protein 7, brain
HES5	Hairy and enhancer of split 5 (Drosophila)

PROM1	Prominin 1
SOX2	SRY (sex determining region Y)-box 2
DCX	Doublecortin
GAD2	Glutamate decarboxylase 2 (pancreatic islets and brain, 65kDa)
SLC32A1	Solute carrier family 32 (GABA vesicular transporter), member 1
ENO1	Enolase 1, (alpha)
MSLN	Mesothelin
FOXG1	Forkhead box G1
OLIG2	Oligodendrocyte lineage transcription factor 2
NKX2-2	NK2 homeobox 2
HAND2	Heart and neural crest derivatives expressed 2
CD79A	CD79a molecule, immunoglobulin-associated alpha
CD3E	CD3e molecule, epsilon (CD3-TCR complex)
PTCRA	Pre T-cell antigen receptor alpha
KRT19	Keratin 19
APOH	Apolipoprotein H (beta-2-glycoprotein I)
DPP4	Dipeptidyl-peptidase 4
MAP3K12	Mitogen-activated protein kinase kinase kinase 12
CDH1	Cadherin 1, type 1, E-cadherin (epithelial)
CDH2	Cadherin 2, type 1, N-cadherin (neuronal)
VTN	Vitronectin
TLN1	Talin 1
WNT5A	Wingless-type MMTV integration site family, member 5A
WNT11	Wingless-type MMTV integration site family, member 11
ITGAV	Integrin, alpha V (vitronectin receptor, alpha polypeptide, antigen CD51)
ITGB5	Integrin, beta 5
PTK2	PTK2 protein tyrosine kinase 2
MAPK12	Mitogen-activated protein kinase 12
MAPK8	Mitogen-activated protein kinase 8
SMAD1	SMAD family member 1
SMAD2	SMAD family member 2

ISL1	ISL LIM homeobox 1
NKX2-5	NK2 homeobox 5
DKK1	Dickkopf homolog 1 (Xenopus laevis)
DKK3	Dickkopf homolog 3 (Xenopus laevis)
SNAI1	Snail homolog 1 (Drosophila)
SNAI2	Snail homolog 2 (Drosophila)
LEFTY2	Left-right determination factor 2
SFRP1	Secreted frizzled-related protein 1
SFRP2	Secreted frizzled-related protein 2
GSC	Goosecoid homeobox
WNT3A	Wingless-type MMTV integration site family, member 3A
GSK3B	Glycogen synthase kinase 3 beta
RHOA	Ras homolog gene family, member A
YY1AP1	YY1 associated protein 1
TAZ	Tafazzin
KLF4	Kruppel-like factor 4 (gut)
MAPK3	Mitogen-activated protein kinase 3
FOXA2	Forkhead box A2
SOX1	SRY (sex determining region Y)-box 1
CER1	Cerberus 1, cysteine knot superfamily, homolog (Xenopus laevis)
LEF1	Lymphoid enhancer-binding factor 1
HDAC3	Histone deacetylase 3
ACTB	Actin, beta
B2M	Beta-2-microglobulin
GAPDH	Glyceraldehyde-3-phosphate dehydrogenase
HPRT1	Hypoxanthine phosphoribosyltransferase 1
RPLP0	Ribosomal protein, large, P0
GDC	
RTC	Reverse Transcription Control
RTC	Reverse Transcription Control
RTC	Reverse Transcription Control
PPC	
PPC	
PPC	

Table 4: Average Delta Ct (ΔCt) values for qPCR analysis using glass controls, patterned glass, 10 kPa hydrogel and patterned 10 kPa hydrogel samples.

	AVG Delta(Ct) (Ct(GOI) - Ave Ct(HKG))			
Symbol	Glass control	Patterned glass	10 kPa	Patterned 10 kPa
DNMT3B	-5.37	-6.16	-6.16	-6.31
GDF3	-0.41	-1.37	-1.59	-1.65
Nanog	8.52	8.26	6.54	6.43
PODXL	-5.20	-4.62	-4.83	-4.85
POU5F1	-5.97	-5.85	-6.02	-6.47
CCDC42	7.59	7.65	7.90	7.41
FGF5	7.25	9.71	7.32	10.26
foxD3	0.81	-0.37	0.23	-0.35
SOX2	-7.85	-4.12	-3.76	-4.04
SOX1	7.97	8.35	9.08	9.84
OTX2	-3.87	-2.43	-2.14	-2.62
ZIC1	2.72	2.18	3.04	2.69
GBX2	2.84	0.94	0.44	0.47
NEUROG2	8.72	9.71	9.97	10.26
BMP4	1.27	2.76	3.12	3.21
SYNE2	-1.10	-1.36	-1.09	-1.44
EMD	-0.71	-0.14	-0.06	-0.18
GATA2	6.63	6.53	7.49	6.59
HAND1	8.54	7.43	9.97	7.03
WDR5	-1.44	-1.79	-1.82	-1.93
MIXL1	2.30	3.52	3.10	2.54
PDGFRA	3.83	2.83	2.70	2.71
RUNX1	6.81	6.01	5.98	6.04
T	1.18	-4.22	-3.60	-3.78
FOXA1	8.74	7.26	7.71	7.62
HDAC1	-2.77	-2.74	-2.83	-2.88
GATA6	3.85	7.73	7.21	5.90
HNF4A	5.24	7.55	6.32	5.73
SOX17	6.98	0.34	-0.15	-0.98
LMNA	0.81	0.73	0.53	0.65
FABP7	8.87	7.82	7.89	7.46
HES5	4.39	7.25	7.65	7.05
PROM1	-2.84	-3.19	-3.11	-3.38
DCX	2.54	2.43	2.00	1.58
GAD2	6.91	4.02	4.05	4.68
SLC32A1	7.65	6.28	5.84	6.25
ENO1	-8.06	-8.12	-8.21	-8.46
MSLN	6.38	6.46	6.73	6.80
FOXG1	3.07	4.07	4.12	3.94
OLIG2	9.03	8.29	9.00	7.28
NKX2-2	7.81	7.99	9.96	7.90
HAND2	9.81	9.71	9.97	8.98
ISL1	7.11	6.84	6.20	6.80
NKX2-5	8.88	8.45	7.43	7.21
CD79A	5.30	5.48	5.27	5.42
CD3E	7.00	6.68	5.90	6.11
PTCRA	7.78	7.35	8.36	10.26
KRT19	-3.65	-3.19	-3.14	-3.44
APOH	9.91	9.71	9.97	10.26
DPP4	1.60	2.53	2.60	2.40
MAP3K12	0.12	0.21	0.60	0.14
CDH1	-8.28	-4.07	-4.04	-4.37
CDH2	-1.46	-2.03	-1.85	-1.99
VTN	6.64	6.01	5.89	5.93
TLN1	-2.31	-2.16	-1.86	-2.53
WNT5A	4.10	2.49	3.74	2.22
WNT11	8.74	9.71	9.97	8.45
ITGAV	-1.68	-1.80	-1.62	-1.76
ITGB5	-3.82	-3.62	-3.96	-4.23
PTK2	-3.27	-3.16	-3.34	-3.58
MAPK12	-0.08	0.33	0.51	0.16
MAPK8	-2.29	-2.17	-2.57	-2.75
SMAD1	-1.04	-1.19	-1.48	-1.67
SMAD2	-2.63	-2.51	-2.42	-2.68
DKK1	4.76	5.48	5.36	4.54
Lefty1	0.32	-1.05	-1.40	-1.50
DKK3	-0.25	0.15	0.05	-0.09
SNAI1	3.42	3.91	3.31	3.46
SNAI2	4.64	3.35	3.21	3.37
LEFTY2	0.84	-0.47	-0.72	-0.77
SFRP1	-7.41	-3.92	-3.96	-3.98
SFRP2	-5.17	-6.12	-6.06	-6.40
GSC	8.57	8.62	9.23	6.36
WNT3A	7.17	9.48	8.49	8.50
GSK3B	-2.03	-1.99	-1.88	-2.30
RHOA	-4.18	-3.93	-3.86	-4.24
YY1AP1	-1.35	-1.08	-0.84	-1.22
TAZ	-0.77	0.01	-0.02	-0.24
KLF4	0.35	0.99	1.42	1.09
MAPK3	-0.63	-0.27	-0.43	-0.47
FOXA2	5.88	4.70	5.51	2.96
CER1	2.87	2.17	3.57	2.61
LEF1	1.75	2.13	2.03	1.67
HDAC3	-3.13	-3.11	-3.15	-3.47
ACTB	-9.84	-9.89	-9.83	-10.04
B2M	-2.84	-2.60	-2.73	-3.15
GAPDH	-7.95	-7.96	-7.98	-8.04
HPRT1	-1.59	-1.57	-1.89	-1.93
RPLP0	-9.40	-8.77	-8.83	-9.26
GDC	9.91	8.98	9.97	10.26

Pluripotency related
Ectoderm
Mesoderm
Endoderm
Ectoderm progenitors
Mesoderm Progenitor
Endoderm Progenitor
EMT and Pathways
Housekeeping Etc

Table 5: Fold change and fold regulation values for qPCR analysis using glass controls, patterned glass, 10 kPa hydrogel and patterned 10 kPa hydrogel samples.

		Fold Change (comparing to control group)		
		Patterned glass	10 kPa	Patterned 10 kPa
Position	Symbol	Fold Change	Fold Change	Fold Change
1	DNMT3B	1.73	1.73	1.92
3	GDF3	1.95	**2.26**	**2.37**
5	Lefty1	**2.59**	**3.29**	**3.54**
7	Nanog	**4.28**	**3.96**	1.20
9	PODXL	0.67	0.78	0.79
11	POU5F1	1.41	1.03	0.92
13	CCDC42	0.96	0.81	1.13
15	FGF5	**0.18**	0.95	**0.12**
17	foxD3	**2.27**	1.49	**2.23**
19	OTX2	**0.37**	**0.30**	**0.42**
21	ZIC1	1.45	0.80	1.02
23	GBX2	**3.75**	**5.28**	**5.16**
49	NEUROG2	0.50	**0.42**	**0.34**
51	BMP4	**0.35**	**0.28**	**0.26**
53	SYNE2	1.20	1.00	1.27
55	EMD	0.68	0.64	0.69
57	GATA2	1.07	0.55	1.03
59	HAND1	**2.15**	**0.37**	**2.84**
61	WDR5	1.27	1.30	1.40
63	MIXL1	**0.43**	0.58	0.85
65	PDGFRA	2.00	**2.19**	**2.18**
67	RUNX1	1.74	1.77	1.71
69	T	**42.19**	**27.38**	**31.06**
71	FOXA1	**2.78**	**2.04**	**2.17**
97	HDAC1	0.98	1.04	1.08
99	GATA6	**0.07**	**0.10**	**0.24**
101	HNF4A	**0.20**	**0.47**	0.71
103	SOX17	**99.46**	**139.65**	**247.88**
105	LMNA	1.06	1.22	1.12
107	FABP7	**2.06**	1.97	**2.65**
109	HES5	**0.14**	**0.10**	**0.16**
111	PROM1	1.27	1.20	1.45
113	SOX2	**0.08**	**0.06**	**0.07**
115	DCX	1.08	1.45	1.94
117	GAD2	**7.42**	**7.26**	**4.70**
119	SLC32A1	**2.59**	**3.51**	**2.65**
145	ENO1	1.05	1.11	1.33
147	MSLN	0.95	0.79	0.75
149	FOXG1	**0.50**	**0.48**	0.54
151	OLIG2	1.67	1.02	**3.36**
153	NKX2-2	0.88	**0.22**	0.94
155	HAND2	1.07	0.90	1.78
157	CD79A	0.89	1.02	0.92
159	CD3E	1.25	**2.15**	1.85
161	PTCRA	1.34	0.67	**0.18**
163	KRT19	0.73	0.70	0.86
165	APOH	1.15	0.97	0.79
167	DPP4	0.53	0.50	0.58
193	MAP3K12	0.94	0.72	0.99
195	CDH1	**0.05**	**0.05**	**0.07**
197	CDH2	1.48	1.31	1.44
199	VTN	1.55	1.68	1.63
201	TLN1	0.90	0.73	1.17
203	WNT5A	**3.05**	1.29	**3.68**
205	WNT11	0.51	**0.43**	1.22
207	ITGAV	1.09	0.96	1.06
209	ITGB5	0.87	1.10	1.33
211	PTK2	0.93	1.05	1.24
213	MAPK12	0.75	0.66	0.84
215	MAPK8	0.92	1.22	1.37
241	SMAD1	1.10	1.35	1.54
243	SMAD2	0.92	0.86	1.03
245	ISL1	1.21	1.88	1.24
247	NKX2-5	1.36	**2.74**	**3.20**
249	DKK1	0.61	0.66	1.17
251	DKK3	0.76	0.81	0.89
253	SNAI1	0.71	1.08	0.98
255	SNAI2	**2.46**	**2.71**	**2.42**
257	LEFTY2	**2.48**	**2.96**	**3.05**
259	SFRP1	**0.09**	**0.09**	**0.09**
261	SFRP2	1.93	1.86	**2.35**
263	GSC	0.97	0.64	**4.63**
289	WNT3A	**0.20**	**0.40**	**0.40**
291	GSK3B	0.97	0.90	1.20
293	RHOA	0.84	0.80	1.04
295	YY1AP1	0.83	0.70	0.91
297	TAZ	0.58	0.59	0.69
299	KLF4	0.64	**0.48**	0.60
301	MAPK3	0.78	0.87	0.89
303	FOXA2	**2.27**	1.30	**7.58**
305	SOX1	0.77	**0.46**	**0.27**
307	CER1	1.63	0.62	1.20
309	LEF1	0.77	0.83	1.06
311	HDAC3	0.98	1.02	1.27
337	ACTB	1.03	0.99	1.15
339	B2M	0.85	0.93	1.24
341	GAPDH	1.01	1.02	1.07
343	HPRT1	0.99	1.24	1.27
345	RPLP0	0.65	0.68	0.91
347	GDC	1.92	0.97	0.79

	Up-Down Regulation (comparing to control group)		
	Patterned glass	10 kPa	Patterned 10 kPa
Symbol	Fold Regulation	Fold Regulation	Fold Regulation
DNMT3B	1.73	1.73	1.92
GDF3	1.95	**2.26**	**2.37**
Lefty1	**2.59**	**3.29**	**3.54**
Nanog	**4.28**	**3.96**	1.20
PODXL	-1.49	-1.29	-1.27
POU5F1	1.41	1.03	-1.08
CCDC42	-1.04	-1.24	1.13
FGF5	**-5.50**	-1.05	**-8.04**
foxD3	**2.27**	1.49	**2.23**
OTX2	**-2.71**	**-3.33**	**-2.38**
ZIC1	1.45	-1.25	1.02
GBX2	**3.75**	**5.28**	**5.16**
NEUROG2	-1.99	**-2.37**	**-2.91**
BMP4	**-2.82**	**-3.61**	**-3.85**
SYNE2	1.20	-1.00	1.27
EMD	-1.48	-1.57	-1.45
GATA2	1.07	-1.81	1.03
HAND1	**2.15**	**-2.69**	**2.84**
WDR5	1.27	1.30	1.40
MIXL1	**-2.33**	-1.74	-1.18
PDGFRA	2.00	**2.19**	**2.18**
RUNX1	1.74	1.77	1.71
T	**42.19**	**27.38**	**31.06**
FOXA1	**2.78**	**2.04**	**2.17**
HDAC1	-1.02	1.04	1.08
GATA6	**-14.75**	**-10.24**	**-4.15**
HNF4A	**-4.98**	**-2.12**	-1.40
SOX17	**99.46**	**139.65**	**247.88**
LMNA	1.06	1.22	1.12
FABP7	**2.06**	1.97	**2.65**
HES5	**-7.24**	**-9.58**	**-6.29**
PROM1	1.27	1.20	1.45
SOX2	**-13.26**	**-17.03**	**-14.01**
DCX	1.08	1.45	1.94
GAD2	**7.42**	**7.26**	**4.70**
SLC32A1	**2.59**	**3.51**	**2.65**
ENO1	1.05	1.11	1.33
MSLN	-1.05	-1.27	-1.33
FOXG1	**-2.00**	**-2.08**	-1.84
OLIG2	1.67	1.02	**3.36**
NKX2-2	-1.14	**-4.44**	-1.07
HAND2	1.07	-1.12	1.78
CD79A	-1.13	1.02	-1.09
CD3E	1.25	**2.15**	1.85
PTCRA	1.34	-1.50	**-5.59**
KRT19	-1.38	-1.43	-1.16
APOH	1.15	-1.04	-1.27
DPP4	-1.89	-2.00	-1.74
MAP3K12	-1.06	-1.39	-1.01
CDH1	**-9.4**	**-11.8**	**-14.99**
CDH2	1.48	1.31	1.44
VTN	1.55	1.68	1.63
TLN1	-1.11	-1.36	1.17
WNT5A	**3.05**	1.29	**3.68**
WNT11	-1.96	**-2.34**	1.22
ITGAV	1.09	-1.05	1.06
ITGB5	-1.14	1.10	1.33
PTK2	-1.08	1.05	1.24
MAPK12	-1.33	-1.51	-1.18
MAPK8	-1.08	1.22	1.37
SMAD1	1.10	1.35	1.54
SMAD2	-1.09	-1.16	1.03
ISL1	1.21	1.88	1.24
NKX2-5	1.36	**2.74**	**3.20**
DKK1	-1.64	-1.51	1.17
DKK3	-1.32	-1.23	-1.12
SNAI1	-1.41	1.08	-1.02
SNAI2	**2.46**	**2.71**	**2.42**
LEFTY2	**2.48**	**2.96**	**3.05**
SFRP1	**-8.18**	**-10.94**	**-10.72**
SFRP2	1.93	1.86	**2.35**
GSC	-1.03	-1.57	**4.63**
WNT3A	**-4.97**	**-2.49**	**-2.52**
GSK3B	-1.03	-1.11	1.20
RHOA	-1.19	-1.25	1.04
YY1AP1	-1.20	-1.43	-1.10
TAZ	-1.72	-1.68	-1.45
KLF4	-1.55	**-2.09**	-1.66
MAPK3	-1.29	-1.16	-1.12
FOXA2	**2.27**	1.30	**7.58**
SOX1	-1.31	**-2.17**	**-3.67**
CER1	1.63	-1.62	1.20
LEF1	-1.29	-1.21	1.06
HDAC3	-1.02	1.02	1.27
ACTB	1.03	-1.01	1.15
B2M	-1.17	-1.08	1.24
GAPDH	1.01	1.02	1.07
HPRT1	-1.01	1.24	1.27
RPLP0	-1.54	-1.48	-1.10
GDC	1.92	-1.04	-1.27

Table 6: Average Delta Ct (ΔCt) values for qPCR analysis using BMP4 induced glass controls, patterned glass, 10 kPa hydrogel and patterned 10 kPa hydrogel samples.

	AVG Delta(Ct) (Ct(GOI) - Ave Ct(HKG))			
**Symbol**	**Glass**	**Patterned glas**	**10 kPa**	**Patterned 10 kPa**
DNMT3B	3.58	4.64	5.22	5.00
GDF3	7.17	4.15	7.92	6.93
Lefty1	10.41	5.93	9.28	8.75
Nanog	15.59	15.46	16.04	17.12
PODXL	3.71	4.27	4.38	5.87
POU5F1	2.99	1.95	2.53	2.88
CCDC42	16.03	15.90	14.34	15.44
FGF5	15.16	16.51	17.14	14.91
foxD3	10.06	10.72	15.03	13.19
OTX2	4.42	5.05	7.13	5.81
ZIC1	11.00	13.48	14.61	14.05
GBX2	14.28	10.28	12.17	10.99
NEUROG2	16.83	18.26	18.16	15.91
BMP4	8.24	6.51	5.19	5.43
SYNE2	8.51	8.41	8.31	8.94
EMD	7.61	8.87	8.74	8.99
GATA2	12.50	9.58	7.26	8.11
HAND1	10.15	8.17	4.31	4.79
WDR5	7.43	6.51	6.70	6.72
MIXL1	8.01	1.53	3.32	2.37
PDGFRA	12.38	7.48	7.68	6.60
RUNX1	15.41	11.78	10.19	10.38
T	9.44	4.56	4.60	4.02
FOXA1	17.96	16.26	17.61	16.66
HDAC1	6.02	5.58	5.25	5.85
GATA6	14.16	5.61	6.13	4.95
HNF4A	17.35	16.71	16.84	16.27
SOX17	15.24	9.12	8.04	6.83
LMNA	9.78	9.11	7.97	8.69
FABP7	18.17	16.45	17.51	17.52
HES5	15.09	18.49	17.46	17.31
PROM1	6.11	5.08	5.49	6.22
SOX2	4.49	7.06	11.58	10.06
DCX	12.47	11.48	11.89	11.91
GAD2	16.47	16.35	18.16	15.83
SLC32A1	14.70	15.26	18.16	16.92
ENO1	1.39	0.67	0.71	0.83
MSLN	13.03	15.21	15.91	16.36
FOXG1	13.74	14.19	15.43	14.68
OLIG2	15.98	16.08	17.37	16.43
NKX2-2	18.17	16.06	15.31	13.88
HAND2	17.01	13.99	12.87	13.48
CD79A	15.11	15.18	14.84	15.09
CD3E	17.33	16.85	17.85	18.06
PTCRA	17.72	17.81	16.92	18.06
KRT19	4.39	3.91	1.77	2.71
APOH	18.17	18.49	18.16	18.06
DPP4	10.51	12.28	12.84	12.13
MAP3K12	9.25	10.63	10.10	10.11
CDH1	4.69	4.00	2.94	4.39
CDH2	7.03	4.70	5.76	5.15
VTN	14.20	15.36	14.82	14.97
TLN1	7.03	6.48	6.37	6.63
WNT5A	10.40	7.39	4.92	4.51
WNT11	17.06	17.70	15.01	15.98
ITGAV	7.64	5.84	5.25	6.45
ITGB5	5.70	3.73	3.68	3.86
PTK2	5.85	5.48	5.42	5.61
MAPK12	9.41	10.09	9.91	8.80
MAPK8	6.44	5.43	5.76	5.75
SMAD1	7.86	6.38	6.38	6.80
SMAD2	6.68	6.07	6.01	6.23
ISL1	9.99	8.02	4.79	6.35
NKX2-5	16.31	13.37	12.15	10.33
DKK1	11.35	5.06	6.20	5.17
DKK3	8.36	8.21	7.40	8.34
SNAI1	11.80	9.77	9.18	8.12
SNAI2	12.94	9.60	6.96	5.60
LEFTY2	11.50	7.19	8.57	8.45
SFRP1	4.26	9.02	8.65	9.13
SFRP2	4.58	3.48	4.66	4.76
GSC	13.37	6.85	10.58	9.33
WNT3A	18.17	16.35	13.99	13.35
GSK3B	7.00	6.15	6.18	6.74
RHOA	4.05	3.85	4.00	4.17
YY1AP1	7.34	7.24	7.26	7.08
TAZ	8.16	9.56	9.01	8.87
KLF4	8.10	9.07	8.95	9.30
MAPK3	8.51	8.86	7.89	7.93
FOXA2	17.97	10.64	12.36	10.02
SOX1	14.19	18.37	18.16	18.06
CER1	11.51	1.29	4.20	2.76
LEF1	9.58	6.93	6.25	5.98
HDAC3	5.70	5.34	5.47	5.58
ACTB	-0.64	-1.31	-1.42	-1.11
B2M	5.86	5.03	4.26	4.90
GAPDH	1.05	0.76	0.70	1.04
HPRT1	7.51	6.75	7.43	7.36
RPLP0	-0.99	-0.73	-0.68	-0.98
GDC	18.17	17.41	18.16	18.06

Table 7: Fold change and fold regulation values for qPCR analysis using BMP4 induced glass controls, patterned glass, 10 kPa hydrogel and patterned 10 kPa hydrogel samples.

	Fold Change Values (comparing to control group-Glass)		
	Glass Patterns	10kPa NP	10kPa Pattern
Symbol	Fold Change	Fold Change	Fold Change
DNMT3B	**0.48**	**0.32**	**0.37**
GDF3	**8.12**	0.59	1.18
Lefty1	**22.40**	**2.19**	**3.17**
Nanog	1.09	0.73	**0.35**
PODXL	0.68	0.63	**0.22**
POU5F1	**2.05**	1.37	1.08
CCDC42	1.09	**3.22**	1.50
FGF5	**0.39**	**0.25**	1.19
foxD3	0.63	**0.03**	**0.11**
OTX2	0.64	**0.15**	**0.38**
ZIC1	**0.18**	**0.08**	**0.12**
GBX2	**16.06**	**4.32**	**9.82**
NEUROG2	**0.37**	**0.40**	1.90
BMP4	**3.33**	**8.28**	**7.03**
SYNE2	1.07	1.15	0.74
EMD	**0.42**	**0.46**	**0.38**
GATA2	**7.59**	**37.79**	**21.05**
HAND1	**3.97**	**57.48**	**41.20**
WDR5	1.89	1.66	1.64
MIXL1	**89.27**	**25.77**	**49.88**
PDGFRA	**29.88**	**25.98**	**55.01**
RUNX1	**12.37**	**37.28**	**32.57**
T	**29.41**	**28.64**	**42.61**
FOXA1	**3.25**	1.27	**2.46**
HDAC1	1.36	1.70	1.13
GATA6	**374.51**	**260.12**	**592.06**
HNF4A	1.56	1.42	**2.12**
SOX17	**69.34**	**146.05**	**339.95**
LMNA	1.59	**3.51**	**2.13**
FABP7	**3.30**	1.58	1.57
HES5	**0.09**	**0.19**	**0.22**
PROM1	**2.05**	1.54	0.93
SOX2	**0.17**	**0.01**	**0.02**
DCX	1.99	1.49	1.48
GAD2	1.09	**0.31**	1.56
SLC32A1	0.68	**0.09**	**0.22**
ENO1	1.65	1.60	1.48
MSLN	**0.22**	**0.14**	**0.10**
FOXG1	0.73	**0.31**	0.52
OLIG2	0.94	**0.38**	0.73
NKX2-2	**4.33**	**7.26**	**19.62**
HAND2	**8.10**	**17.63**	**11.59**
CD79A	0.96	1.21	1.01
CD3E	1.40	0.70	0.60
PTCRA	0.95	1.75	0.79
KRT19	1.40	**6.17**	**3.20**
APOH	0.80	1.01	1.08
DPP4	**0.29**	**0.20**	**0.33**
MAP3K12	**0.38**	0.55	0.55
CDH1	1.62	**3.37**	1.23
CDH2	**5.02**	**2.42**	**3.68**
VTN	**0.45**	0.65	0.59
TLN1	1.46	1.58	1.32
WNT5A	**8.03**	**44.49**	**59.28**
WNT11	0.64	**4.13**	**2.11**
ITGAV	**3.47**	**5.24**	**2.27**
ITGB5	**3.92**	**4.07**	**3.58**
PTK2	1.29	1.35	1.18
MAPK12	0.62	0.71	1.52
MAPK8	**2.01**	1.60	1.61
SMAD1	**2.79**	**2.80**	**2.09**
SMAD2	1.53	1.59	1.37
ISL1	**3.93**	**36.76**	**12.54**
NKX2-5	**7.65**	**17.89**	**63.31**
DKK1	**78.37**	**35.62**	**72.37**
DKK3	1.11	1.95	1.01
SNAI1	**4.07**	**6.12**	**12.82**
SNAI2	**10.19**	**63.26**	**162.86**
LEFTY2	**19.88**	**7.63**	**8.29**
SFRP1	**0.04**	**0.05**	**0.03**
SFRP2	**2.14**	0.95	0.88
GSC	**91.66**	**6.90**	**16.44**
WNT3A	**3.54**	**18.09**	**28.22**
GSK3B	1.80	1.76	1.19
RHOA	1.14	1.03	0.92
YY1AP1	1.07	1.06	1.20
TAZ	**0.38**	0.55	0.61
KLF4	0.51	0.56	**0.44**
MAPK3	0.78	1.53	1.50
FOXA2	**160.08**	**48.81**	**246.83**
SOX1	**0.06**	**0.06**	**0.07**
CER1	**1191.87**	**159.11**	**430.14**
LEF1	**6.28**	**10.09**	**12.12**
HDAC3	1.29	1.17	1.09
ACTB	1.60	1.72	1.39
B2M	1.77	**3.01**	1.94
GAPDH	1.22	1.27	1.00
HPRT1	1.69	1.05	1.10
RPLP0	0.83	0.81	1.00
GDC	1.70	1.01	1.08

	Up-Down Regulation (comparing to control group)		
	Glass Pattern	10kPa NP	10kPa Pattern
Symbol	Fold Regulation	Fold Regulation	Fold Regulation
DNMT3B	**-2.08**	**-3.11**	**-2.67**
GDF3	**8.12**	-1.68	1.18
Lefty1	**22.40**	**2.19**	**3.17**
Nanog	1.09	-1.37	**-2.90**
PODXL	-1.47	-1.59	**-4.46**
POU5F1	**2.05**	1.37	1.08
CCDC42	1.09	**3.22**	1.50
FGF5	**-2.55**	**-3.95**	1.19
foxD3	-1.58	**-31.20**	**-8.74**
OTX2	-1.55	**-6.54**	**-2.62**
ZIC1	**-5.59**	**-12.28**	**-8.29**
GBX2	**16.06**	**4.32**	**9.82**
NEUROG2	**-2.68**	**-2.51**	1.90
BMP4	**3.33**	**8.28**	**7.03**
SYNE2	1.07	1.15	-1.34
EMD	**-2.41**	**-2.19**	**-2.61**
GATA2	**7.59**	**37.79**	**21.05**
HAND1	**3.97**	**57.48**	**41.20**
WDR5	1.89	1.66	1.64
MIXL1	**89.27**	**25.77**	**49.88**
PDGFRA	**29.88**	**25.98**	**55.01**
RUNX1	**12.37**	**37.28**	**32.57**
T	**29.41**	**28.64**	**42.61**
FOXA1	**3.25**	1.27	**2.46**
HDAC1	1.36	1.70	1.13
GATA6	**374.51**	**260.12**	**592.06**
HNF4A	1.56	1.42	**2.12**
SOX17	**69.34**	**146.05**	**339.95**
LMNA	1.59	**3.51**	**2.13**
FABP7	**3.30**	1.58	1.57
HES5	**-10.54**	**-5.15**	**-4.64**
PROM1	**2.05**	1.54	-1.08
SOX2	**-5.92**	**-135.61**	**-47.26**
DCX	1.99	1.49	1.48
GAD2	1.09	**-3.22**	1.56
SLC32A1	-1.47	**-10.96**	**-4.64**
ENO1	1.65	1.60	1.48
MSLN	**-4.52**	**-7.34**	**-10.04**
FOXG1	-1.37	**-3.23**	-1.93
OLIG2	-1.07	**-2.61**	-1.37
NKX2-2	**4.33**	**7.26**	**19.62**
HAND2	**8.10**	**17.63**	**11.59**
CD79A	-1.05	1.21	1.01
CD3E	1.40	-1.43	-1.66
PTCRA	-1.06	1.75	-1.26
KRT19	1.40	**6.17**	**3.20**
APOH	-1.25	1.01	1.08
DPP4	**-3.43**	**-5.04**	**-3.07**
MAP3K12	**-2.61**	-1.81	-1.82
CDH1	1.62	**3.37**	1.23
CDH2	**5.02**	**2.42**	**3.68**
VTN	**-2.23**	-1.53	-1.70
TLN1	1.46	1.58	1.32
WNT5A	**8.03**	**44.49**	**59.28**
WNT11	-1.56	**4.13**	**2.11**
ITGAV	**3.47**	**5.24**	**2.27**
ITGB5	**3.92**	**4.07**	**3.58**
PTK2	1.29	1.35	1.18
MAPK12	-1.61	-1.42	1.52
MAPK8	**2.01**	1.60	1.61
SMAD1	**2.79**	**2.80**	**2.09**
SMAD2	1.53	1.59	1.37
ISL1	**3.93**	**36.76**	**12.54**
NKX2-5	**7.65**	**17.89**	**63.31**
DKK1	**78.37**	**35.62**	**72.37**
DKK3	1.11	1.95	1.01
SNAI1	**4.07**	**6.12**	**12.82**
SNAI2	**10.19**	**63.26**	**162.86**
LEFTY2	**19.88**	**7.63**	**8.29**
SFRP1	**-27.21**	**-21.06**	**-29.40**
SFRP2	**2.14**	-1.06	-1.13
GSC	**91.66**	**6.90**	**16.44**
WNT3A	**3.54**	**18.09**	**28.22**
GSK3B	1.80	1.76	1.19
RHOA	1.14	1.03	-1.09
YY1AP1	1.07	1.06	1.20
TAZ	**-2.64**	-1.81	-1.63
KLF4	-1.96	-1.80	**-2.29**
MAPK3	-1.28	1.53	1.50
FOXA2	**160.08**	**48.81**	**246.83**
SOX1	**-18.13**	**-15.65**	**-14.63**
CER1	**1191.87**	**159.11**	**430.14**
LEF1	**6.28**	**10.09**	**12.12**
HDAC3	1.29	1.17	1.09
ACTB	1.60	1.72	1.39
B2M	1.77	**3.01**	1.94
GAPDH	1.22	1.27	1.00
HPRT1	1.69	1.05	1.10
RPLP0	-1.20	-1.24	-1.00
GDC	1.70	1.01	1.08
